# Demographic risk factors for adverse birth outcomes in Puerto Rico in the PROTECT cohort

**DOI:** 10.1371/journal.pone.0217770

**Published:** 2019-06-13

**Authors:** Kelly K. Ferguson, Zaira Rosario, Thomas F. McElrath, Carmen Vélez Vega, José F. Cordero, Akram Alshawabkeh, John D. Meeker

**Affiliations:** 1 Epidemiology Branch, National Institute of Environmental Health Sciences, Research Triangle Park, North Carolina, United States of America; 2 Department of Environmental Health Sciences, University of Michigan School of Public Health, Ann Arbor, Michigan, United States of America; 3 University of Puerto Rico Graduate School of Public Health, San Juan, Puerto Rico; 4 Division of Maternal-Fetal Medicine, Brigham and Women’s Hospital, Harvard Medical School, Boston, Massachusetts, United States of America; 5 Epidemiology and Biostatistics Department, College of Public Health, University of Georgia, Athens, Georgia, United States of America; 6 College of Engineering, Northeastern University, Boston, Massachusetts, United States of America; Johns Hopkins School of Public Health, UNITED STATES

## Abstract

Preterm birth is a major public health problem, especially in Puerto Rico where the rates are among the highest observed worldwide, reaching 18% in 2011. The Puerto Rico Testsite for Exploring Contamination Threats (PROTECT) study is an ongoing investigation of environmental factors that contribute to this condition. In the present analysis, we sought to examine common risk factors for preterm birth and other adverse birth outcomes which have not been characterized previously in this unique population. Pregnant women from the PROTECT cohort are recruited from the heavily contaminated Northern coast of the island of Puerto Rico and are free of pre-existing conditions like diabetes. We examined associations between basic demographic, behavioral (e.g., tobacco and alcohol use), and pregnancy (e.g., season and year of delivery) characteristics as well as municipality of residence in relation to preterm birth (<37 weeks gestation), postterm birth (≥41 weeks gestation), and small and large for gestational age in univariate and multivariate logistic regression models. Between 2011 and 2017, 1028 live singleton births were delivered as part of the PROTECT cohort. Of these, 107 (10%) were preterm. Preterm birth rates were higher among women with low socioeconomic status, as indicated by education level and income, and among women with high pre-pregnancy body mass index (BMI). Odds ratios of small for gestational age delivery were higher for women who reported tobacco use in pregnancy and lower for women who delivered in the hurricane and dengue season (July-October). Overall, in pregnant women residing in Puerto Rico, socioeconomic status was associated with preterm birth but few other factors were associated with this or other adverse outcomes of pregnancy. Research to understand environmental factors that could be contributing to the preterm birth epidemic in Puerto Rico is necessary.

## Introduction

Preterm birth is a global health problem that has major public health and economic consequences [1]. In Puerto Rico, the rate of preterm birth is particularly high. In 2011, the island of Puerto shared the highest rate of preterm birth in the United States with Mississippi at 18% [2], which is also among the highest rates worldwide [3]. By 2017, this decreased considerably to 11% [4]. However, this still ranks among the highest rates of preterm birth, especially for highly developed countries, but also in the world [3, 5]. Thus, identifying factors contributing to preterm birth in Puerto Rico deserves special attention.

The Puerto Rico Testsite for Exploring Contamination Threats (PROTECT) cohort study was designed in 2010 to investigate the etiology of preterm birth on the island. This question is of critical public health importance because there is no explanation to date for the higher rates of preterm birth observed in Puerto Rico. Additionally, this study particularly aims to investigate environmental contaminants in the etiology of preterm birth because of the high density of Superfund waste sites located on the island. However, these factors cannot be examined without first understanding how the demographic and behavioral characteristics that are well-established predictors of preterm birth in the literature, including maternal age, socioeconomic factors, tobacco use in pregnancy, maternal body mass index (BMI), etc., are associated with preterm birth in this study population [6–9]. The associations between these characteristics and preterm birth have never been examined among pregnant women residing in Puerto Rico.

Thus, the objective of the present analysis was to establish whether or not demographic and behavioral risk factors that have been traditionally associated with preterm birth also exist in this study population, and to see whether these factors appear to be driving the higher preterm birth rates observed on the island. Additionally, since recruitment into PROTECT is ongoing, these data also establish rates of preterm birth within our study population prior to the devastation of Hurricanes Irma and Maria (August-September 2017) and their continued aftermath.

## Materials and methods

PROTECT study participants are recruited on the island’s heavily contaminated northern coast at two collaborating hospitals and five nearby health clinics. All participants are recruited from clinics located in Camuy, Lares, Morovis, Quebradillas, and Ciales, and intend to deliver at one of the two collaborating hospitals: Manatí Medical Center and Arecibo’s Cayetano Coll y Toste Hospital. Pregnant women visiting the clinics or hospitals are recruited at approximately 14 weeks gestation and are eligible for inclusion in the study if they are between the ages of 18 and 40 years, reside in a municipality in the Northern karst region of the island, did not use oral contraceptives for at least three months prior to becoming pregnant, did not use *in vitro* fertilization to become pregnant, and were free of known medical or obstetrical complications, including pre-existing diabetes [10]. If eligible, women are invited to participate and asked to fill out an initial screening form to collect brief information on demographic characteristics and estimated date of last menstrual period (LMP). The study was described in detail to all participants, and written informed consent was obtained prior to study enrollment.

Following the initial screening, women are invited to participate in three study visits. The first study visit is targeted for 20±2 weeks gestation and is performed in the clinic, at which point women are asked to provide urine and blood samples and fill out a questionnaire that collects information on demographic characteristics, personal care product use over the previous 48 hours, current and previous pregnancy complications, and physical activity. The second visit is targeted for 24±2 weeks and is performed in the participant’s home. At this time point a urine sample is collected, and questionnaires are administered to collect information on behavioral characteristics, medication use, physical activity, stressful life experiences, product and pesticide use, and dietary intake patterns (i.e., food frequency questionnaire). The third visit, targeted for 28±2 gestation, is also performed in the clinic and collects the same samples and information as the first visit, but has additional questionnaires to collect information on psychological stress and social support in pregnancy. At delivery, detailed information is collected on the pregnancy outcome along with birth weight and other newborn measurements. The research protocol for PROTECT was approved by the Ethics and Research Committees of the University of Puerto Rico and participating clinics, the University of Michigan, and Northeastern University.

In order to the understand the basic risk factors for adverse birth outcomes in women residing in Puerto Rico, in the present analysis we examined associations with demographic, behavioral, and pregnancy characteristics among women who delivered live singleton births in PROTECT up until the month of Hurricane Irma (August 2017).

### Demographic characteristics and geographic location

For the present analysis, we examined demographic, behavioral, and pregnancy characteristics as well as municipality of residence of the study population in relation to adverse birth outcomes. Most demographic factors were abstracted from initial screening or first visit questionnaires that were collected early in pregnancy (targeted at 14 and 20 weeks of gestation, respectively; S1 File). These included: maternal age at enrollment (years); pre-pregnancy BMI in kg/m^2^ calculated from self-reported pre-pregnancy weight and height at the first study visit and categorized as <25 kg/m^2^ (normal or underweight), 25–30 kg/m^2^ (overweight), and >30 kg/m^2^ (obese); total household income in US dollars (assessed by questionnaire with the following categories: <$20k; $20-30k; $30-40k; $40-50k; $50-75k; $75-100k; $100-200k; >$200k); maternal education level (<High school, High school or equivalent, Some college or technical school, and College graduate or above); employment status; and marital status. Alcohol use was assessed at the first study visit with a question about the last time the subject consumed one alcoholic beverage. If the participant reported that she does not drink alcohol, or that the previous drink was prior to pregnancy, then she was categorized as not using alcohol in pregnancy. Otherwise, she was coded as using alcohol in pregnancy. Information on smoking was also recorded at the first study visit. Participants were coded as not using tobacco in pregnancy if they reported never smoking or if they reported ever smoking but also reported quitting ≥6 months ago or prior to pregnancy. Otherwise, they were coded as smoking during pregnancy. Participants with missing information on tobacco or alcohol use were treated as missing (i.e., they were not recoded as non-users). Number of pregnancies prior to the current pregnancy (gravidity) and number of prior births (parity) for each participant were obtained from the first study visit questionnaire as well.

Municipality of residence was self-reported on the initial screening questionnaire and used as an indicator of geographic location. For municipalities with fewer than 50 participants, the results were condensed into an “Other” category.

### Pregnancy characteristics and outcomes

Characteristics of the current pregnancy were obtained from medical records after delivery. These data included: mode of delivery (vaginal or C-section); infant sex (male or female); and date of delivery that was used to calculate final gestational age, season of delivery (July to October, corresponding to hurricane and dengue seasons, or November-June), and year of delivery (2011 to 2017).

Gestational age at delivery was assessed using self-reported date of LMP, collected at the initial screening, in combination with first ultrasound estimates of gestational age [11]. Preterm birth was defined as delivery before 37 weeks completed gestation, and postterm birth was defined as delivery at or after 41 weeks completed gestation. Birth weight was recorded in grams. Z-scores for birth weight were calculated based on gestational age at the time of delivery and fetal sex using the INTERGROWTH-21^st^ standard [12]. Small for gestational age (SGA) was assigned based birth weight <10^th^ percentile and large for gestational age (LGA) was assigned based on birth weight >90^th^ percentile for gestational age.

As secondary analyses, we also investigated other adverse pregnancy outcomes including preeclampsia, gestational diabetes, low birth weight (<2500 grams), macrosomia (>4000 grams), as well as miscarriage (loss before 20 weeks of gestation) and stillbirth (loss after 20 weeks of gestation). Low birth weight and macrosomia were defined based on recorded birth weight at delivery and other adverse pregnancy outcomes were defined based on diagnosis in medical record by attending physician.

### Statistical analysis

All analyses were performed using SAS version 9.4 (Cary, NC). First, we examined distributions of demographic, behavioral, and pregnancy characteristics within the study population. Second, we examined mean and standard deviation gestational age (weeks) and birth weight (grams) at delivery overall and by population characteristics listed above as well as municipality of residence. We tested for differences in gestational age at delivery and birth weight between categorical variables with linear regression models. For differences by municipality, we compared outcomes within each municipality to those in all other municipalities using T-Tests without assuming equal variances. Third, we examined associations between each characteristic and categorical pregnancy outcomes in models that were not mutually adjusted. We calculated odds ratios (OR) for preterm or postterm birth in separate models. The OR for preterm birth was calculated with postterm births excluded and vice versa. We similarly calculated OR for SGA and LGA in separate models. Finally, we created a multivariate model for each outcome to mutually adjust for all risk factors in order to contextually interpret our findings.

## Results

**Fig 1:**
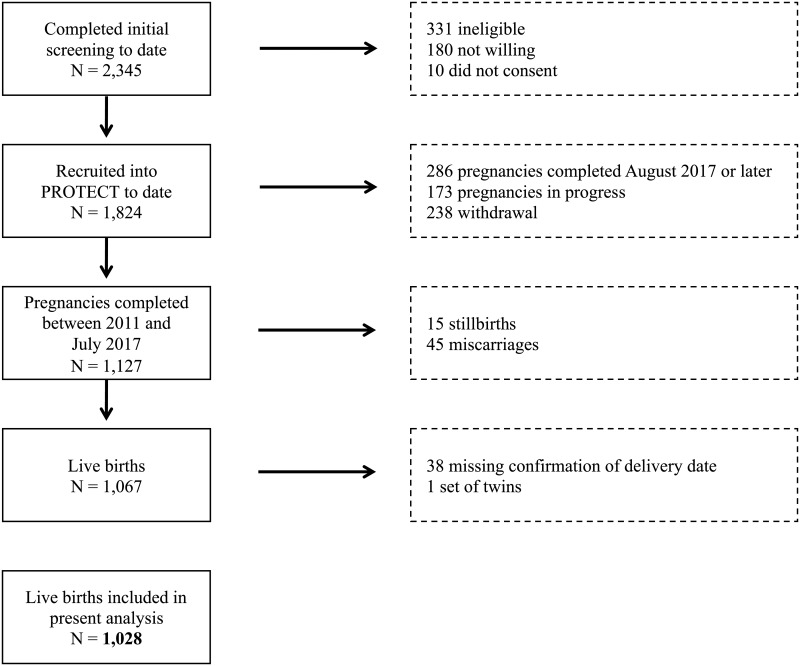
Participants from the Puerto Rico Test site for Exploring Contamination Threats (PROTECT) pregnancy cohort.

The present analysis includes 1028 singleton live births from PROTECT participants who delivered between August 2011 and the end of July of 2017 and who had confirmed delivery and a reported date of LMP on the initial screening form (Fig 1). Ultrasounds for confirming estimated gestational age based on LMP were available for 782 participants (76%). The American College of Obstetricians and Gynecologists (ACOG) recommends redating LMP-based estimated date of delivery if the estimates of gestational age based on LMP differ from ultrasound estimates by a certain number of days, depending on the time of the scan [11]. Table A in S2 File shows the criteria suggested by ACOG for redating based on gestational age at ultrasound, and the number of participants from PROTECT that fell into each category. Most participants who had ultrasounds available for dating had them performed before 14 weeks gestation (n = 693). Overall, gestational age was changed from the LMP estimate to the ultrasound estimate for 171 pregnancies [11], and the change in final gestational age was minimal (mean = 0.25 weeks, standard deviation = 0.13 weeks).

Demographic and pregnancy characteristics of the study population are presented in Table 1. The mean maternal age at enrollment in the study population was 26 years among all pregnancies (range 18–41), and 25 years among women having their first birth. Approximately half (54%) of the population was underweight or normal weight prior to pregnancy (BMI < 25 kg/m^2^), with a population median (range) of 24.1 kg/m^2^ (14.7–50.2 kg/m^2^). Household income was condensed into the following categories based on small sample size in the higher income categories: <$20k; $20-40k; and >$40k. Forty percent of the study population had a household income below $20k per year; however, they were well-educated with 43 percent having a college degree or higher. Sixty-one percent of the study population was employed, and most participants were married or living together and did not smoke or drink alcohol during pregnancy (79, 97, and 92%, respectively). Finally, 59% of the population had more than one previous pregnancy and 51% had one or more previous births.

**Table 1:** Characteristics of mothers with singleton live births in the PROTECT cohort, 2011–2017 (N = 1028).

	n (%)	Mean (SD) GA at delivery (weeks)	Mean (SD) birth weight (grams)
Age at enrollment (years)			
18–24 years	393 (38)	38.7 (2.09)	3069 (512)
25–29 years	316 (31)	38.9 (1.91)	3219 (507)*
30–34 years	209 (20)	38.7 (2.48)	3162 (587)*
>35 years	109 (11)	38.9 (1.76)	3198 (597)*
*missing*	1 (0)	39.0 (.)	3459 (.)
Pre-pregnancy body mass index (kg/m^2^)			
<25	550 (54)	38.9 (1.91)	3140 (505)
25–30	266 (26)	38.9 (1.86)	3210 (561)
>30	166 (16)	38.4 (2.81)*	3118 (627)
*missing*	46 (4)	38.2 (2.16)*	2986 (416)*
Household income			
<$20,000	411 (40)	38.5 (2.42)	3070 (561)
$20,000-$40,000	264 (26)	39.1 (2.02)*	3184 (513)*
>$40,000	209 (20)	39.0 (1.66)	3259 (549)
*missing*	144 (14)	38.7 (1.63)	3146 (469)
Education level			
<High school	77 (7)	38.0 (2.96)	3023 (601)
High school or equivalent	132 (13)	38.4 (2.18)*	3102 (494)*
Some college or technical school	366 (36)	38.7 (2.13)	3133 (509)
College degree or higher	441 (43)	39.1 (1.81)*	3200 (561)
*missing*	12 (1)	38.8 (1.27)	3003 (479)
Employment status			
Unemployed	384 (37)	38.5 (2.06)	3113 (504)
Employed	629 (61)	39.0 (2.10)*	3178 (557)
*missing*	15 (1)	38.0 (1.98)	2814 (491)*
Marital status			
Single	209 (20)	38.7 (1.93)	3128 (471)
Married or cohabitating	810 (79)	38.8 (2.14)	3157 (555)
*missing*	9 (1)	38.4 (0.90)	2857 (466)
Smoking during pregnancy			
No	1000 (97)	38.8 (2.11)	3152 (540)
Yes	18 (2)	39.2 (1.20)	3068 (488)
*missing*	10 (1)	38.5 (0.89)	2934 (503)
Alcohol use during pregnancy			
No	949 (92)	38.8 (2.07)	3147 (541)
Yes	64 (6)	38.8 (2.51)	3186 (507)
*missing*	15 (1)	38.6 (1.46)	3052 (584)
Gravidity			
0–1 previous pregnancy	415 (40)	39.0 (2.22)	3122 (557)
>1 previous pregnancy	603 (59)	38.6 (2.00)*	3171 (527)
*missing*	10 (1)	38.7 (1.11)	2900 (460)
Parity			
No previous births	492 (48)	39.0 (2.14)	3130 (552)
One or more previous birth	526 (51)	38.6 (2.04)*	3171 (527)
*missing*	10 (1)	38.7 (1.11)	2900 (460)
*Final gestational age at delivery*			
*<37 weeks gestation*	107 (10)	34.2 (2.90)	2384 (691)
*≥37 weeks and <41 weeks gestation*	858 (83)	39.2 (0.96)*	3217 (444)*
*≥41 weeks gestation*	63 (6)	41.4 (0.43)*	3403 (490)*
*Birth weight*			
*Small for gestational age*	96 (9)	39.0 (2.07)	2411 (454)
*Appropriate for gestational age*	799 (78)	38.9 (1.79)*	3148 (415)*
*Large for gestational age*	98 (10)	38.5 (2.34)*	3901 (434)*
*missing*	35 (3)	36.4 (4.73)*	879 (.)
*Mode of delivery*			
*Vaginal*	531 (52)	38.9 (1.95)	3136 (504)
*C-section*	470 (46)	38.8 (2.13)	3162 (577)
*missing*	27 (3)	37.3 (3.36)*	(.)
*Infant sex*			
*Male*	527 (51)	38.8 (2.09)	3197 (543)
*Female*	473 (46)	38.8 (1.97)	3094 (530)*
*missing*	28 (3)	37.3 (3.30)*	(.)
*Season of delivery*			
*November—June*	688 (67)	38.8 (2.09)	3143 (534)
*July—October*	340 (33)	38.7 (2.10)	3159 (550)
*Year of delivery*			
*2011*	26 (3)	38.6 (2.20)	3034 (593)
*2012*	170 (17)	38.7 (1.81)	3128 (493)
*2013*	201 (20)	38.8 (1.83)	3127 (467)
*2014*	142 (14)	38.8 (2.22)	3164 (574)
*2015*	180 (18)	38.7 (2.38)	3140 (574)
*2016*	226 (22)	38.7 (2.34)	3147 (584)
*2017*	83 (8)	39.3 (1.51)	3288 (493)*

*p<0.05 for difference from reference (first level, or 2014 for year of delivery). p for trend over time (2011 to 2017) = 0.21 for gestational age and 0.09 for birth weight. Notes: Missing SD values “(.)” reflect cells where only one participant contributed data. For mode of delivery and infant sex, all participants who were missing data were also missing birth weight at delivery. Abbreviations: SD, standard deviation; GA, gestational age.

The median gestational age at delivery in the overall population was 39.1 weeks (range 23.3 to 42.7 weeks), with 10% of singleton live births occurring preterm and 6% occurring postterm (Table 1). The median birth weight was 3175 grams (range 595 to 4904 grams), with 9% of babies born SGA and 8.5% born LGA. Forty babies (4%) were born with macrosomia while 89 (9%) were born low birth weight. Of women who delivered by C-section (46%), most occurred because of failure to progress (28%) or previous C-section (31%). Women who delivered preterm, but not postterm, SGA, or LGA, were more likely to have a C-section.

Participants from PROTECT resided in 21 different municipalities. At least 50 participants resided in each of the following municipalities: Arecibo, Barceloneta, Camuy, Ciales, Manatí, Morovis, and Vega Baja. Other municipalities had fewer than 50 participants and were combined into the “Other” category, and included: Aguadilla, Corozal, Dorado, Florida, Hatillo, Isabela, Lares, Orocovis, Quebradillas, San Sebastián, Toa Alta, Toa Baja, Utuado, and Vega Alta. Distributions of gestational age at delivery as well as birth weight by municipality of residence are presented in Table 2. Compared to the overall population, mothers residing in Barceloneta, Camuy, and Vega Baja had longer gestations. Residents of Ciales and Morovis, however, had lower gestational duration (38.1 and 38.4 weeks, respectively) compared to the overall population (38.8 weeks). Additionally, residents of Morovis gave birth to babies of lower weight (mean 3055 grams) compared to overall (3148 grams).

**Table 2:** Characteristics of pregnancies by municipality of residence: Mean (SD) or n (%).

	Overall	Arecibo	Barceloneta	Camuy	Ciales	Manatí	Morovis	Vega Baja	Other
Final GA at delivery									
(weeks)	38.8 (2.09)	39.0 (1.99)	39.1 (1.42)*	39.3 (1.28)*	38.1 (2.72)*	39.0 (2.08)	38.4 (2.18)*	39.1 (1.68)*	38.8 (2.12)
<37	107 (10.4)	11 (8.9)	3 (3.8)	3 (4.8)	20 (16.0)	10 (8.0)	26 (15.5)	8 (8.2)	26 (10.5)
≥37 and <41	858 (83.5)	103 (83.7)	72 (90.0)	55 (87.3)	102 (81.6)	106 (84.8)	133 (79.2)	82 (84.5)	205 (83.0)
≥41	63 (6.1)	9 (7.3)	5 (6.3)	5 (7.9)	3 (2.4)	9 (7.2)	9 (5.4)	7 (7.2)	16 (6.5)
Birth weight									
(grams)	3148 (539)	3196 (617)	3122 (429)	3132 (470)	3052 (598)	3190 (496)	3055 (522)*	3214 (452)	3204 (570)
SGA	96 (9.3)	14 (11.4)	6 (7.5)	10 (15.9)	12 (9.6)	9 (7.2)	19 (11.3)	8 (8.2)	18 (7.3)
AGA	799 (77.7)	90 (73.2)	71 (88.8)	46 (73.0)	94 (75.2)	101 (80.8)	133 (79.2)	76 (78.4)	188 (76.1)
LGA	98 (9.5)	13 (10.6)	2 (2.5)	4 (6.3)	15 (12.0)	12 (9.6)	10 (6.0)	12 (12.4)	30 (12.1)
*missing*	35 (3.4)	6 (4.9)	1 (1.3)	3 (4.8)	4 (3.2)	3 (2.4)	6 (3.6)	1 (1.0)	11 (4.5)

*p<0.05 for T-Test comparing participants from one municipality to all others. Abbreviations: SD, standard deviation; GA, gestational age; SGA, small for gestational age; AGA, appropriate for gestational age; LGA, large for gestational age.

**Fig 2:**
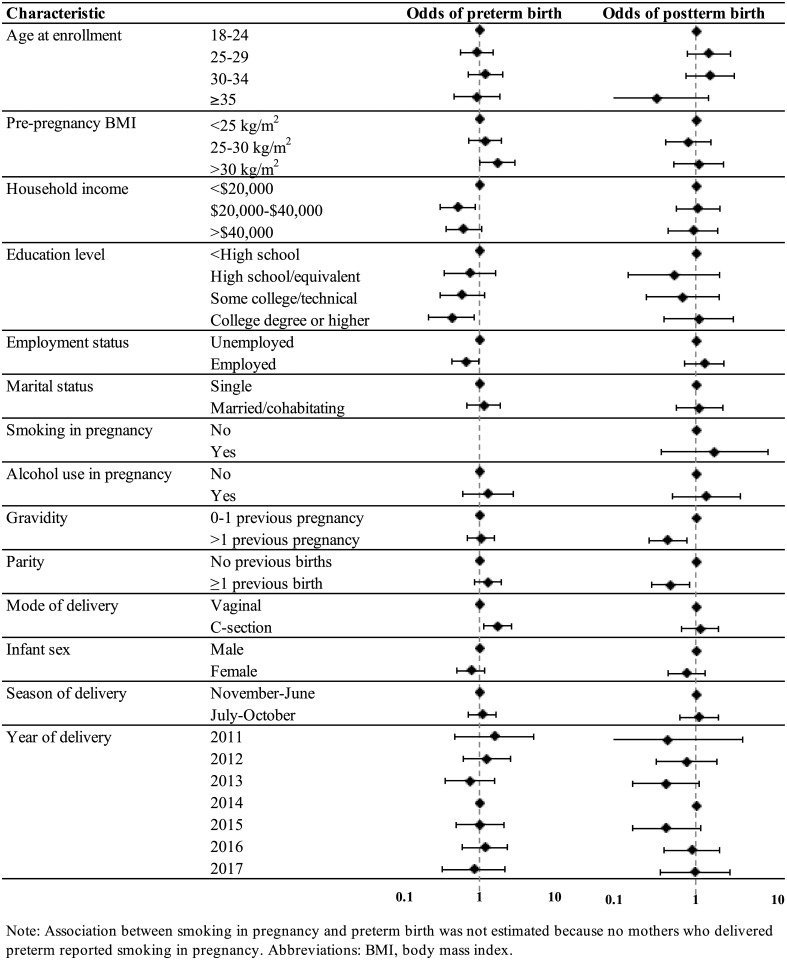
Odds ratios and 95% confidence intervals for preterm and postterm birth in association with demographic and pregnancy characteristics in the PROTECT cohort 2011–2017.

Associations between demographic and pregnancy factors and preterm birth and postterm birth are displayed in Fig 2, with sample sizes by category and effect estimates presented in Tables B and C in S2 File. Socioeconomic factors, including household income, education level, and employment status, showed the most consistent associations with preterm birth. These results were also observed in mutually adjusted models (Table D in S2 File); however, the association between education level and preterm birth was the most pronounced. Adjusted odds ratios for mothers with a college degree or higher were markedly reduced compared to women with a high school degree or equivalent (adjusted OR = 0.36, 95% confidence interval [CI] = 0.16, 0.82). Women with a preterm birth were more likely to have a C-section than a vaginal delivery (OR = 1.81, 1.14, 2.87). Pre-pregnancy BMI was also associated with preterm birth (OR = 1.73, 95% CI = 1.01, 2.96, for women >30 kg/m^2^ compared to women <25 kg/m^2^), and the association was similar in mutually adjusted models (adjusted OR = 1.65, 95% CI = 0.94, 2.88). Postterm birth, on the other hand, was only associated with parity and gravidity (Fig 2 and Table C in S2 File), and these associations were attenuated in mutually adjusted models (Table E in S2 File).

**Fig 3:**
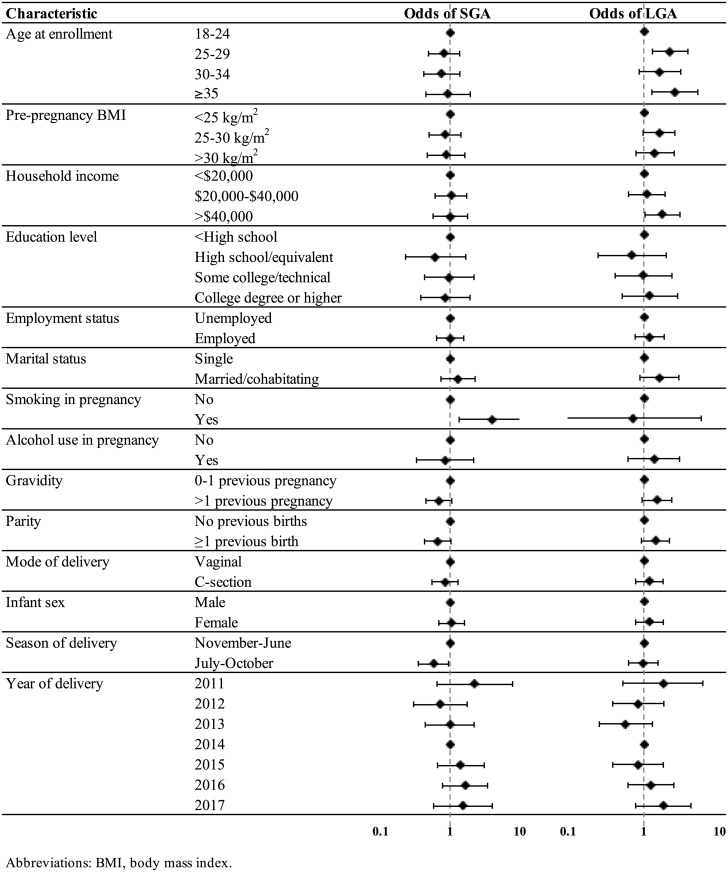
Odds ratios and 95% confidence intervals for delivery of small for gestational age (SGA) and large for gestational age (LGA) newborns in association with demographic and pregnancy characteristics in the PROTECT cohort 2011–2017.

Associations between demographic and pregnancy factors and SGA and LGA are presented in Fig 3, with sample sizes by category and effect estimates presented in S2 File Tables F and G. Smoking was strongly associated with SGA, with smokers having an increased OR of SGA compared to non-smokers (OR = 3.98, 95% CI = 1.35, 11.7). This association remained, and was even greater in magnitude, in mutually adjusted models as well (aOR = 5.61, 95% CI = 1.73, 18.2; Table H in S2 File). Delivery between the hurricane and dengue months of July-October, i.e., gestation outside of this period, was associated with reduced odds of SGA in unadjusted (OR = 0.58, 95% CI = 0.35, 0.95) and adjusted (aOR = 0.61, 95% CI = 0.37, 1.01) models. For LGA, we only observed maternal age and family income to be associated with the outcome, and only age was associated with LGA in adjusted models (Table I in S2 File), where mothers ages 25–29 (aOR = 2.40, 95% CI = 1.30, 4.44), 30–34 (aOR = 1.79, 95% CI = 0.86, 3.70), and ≥35 (aOR = 2.60, 1.18, 5.74) had higher odds of delivering LGA compared to mothers <25.

Preeclampsia occurred in 3% (n = 33) mothers, 15 of whom gave birth to preterm infants. The mean (SD) gestational age at delivery among women who had preeclampsia was 36.4 (3.25) weeks, and the mean (SD) birth weight in grams at delivery was 2,500 (904). Gestational diabetes occurred in 2.3% (n = 24) of mothers. Of these, three gave birth to preterm infants and one experienced gestational diabetes in combination with preeclampsia. Among infants born to women with gestational diabetes, the mean gestational age (38.9 weeks) and birth weight (3,215) did not differ dramatically from the rest of the study population.

## Discussion

Driven by the high preterm birth rates observed in Puerto Rico, the objective of the PROTECT study is to investigate the origins of this disease. The present analysis takes the first step toward understanding this problem by examining risk factors that have been associated with preterm birth in other study populations, and investigating them for the first time among pregnant women in Puerto Rico. We observed that low socioeconomic status, particularly as indicated by education level, was associated with increased risk of preterm birth, as was high BMI. On the other hand, maternal tobacco use in pregnancy and delivery during the hurricane and dengue season (i.e., gestation largely occurring outside of this period) were associated with increased risk of delivering a baby small for gestational age. These findings are consistent with results from other cohort studies in the US and worldwide, but, importantly, have not been previously shown in a population of pregnant women residing in Puerto Rico.

The preterm birth rate in our study population (10% overall, 10.1% in 2014) is lower than what has been reported recently in the March of Dimes report cards (15.1% in 2014). This is likely due to our exclusion criteria which omitted women with pre-existing conditions that are risk factors for preterm birth, including diabetes as well as IVF [9, 13]. Women with preexisting conditions, such as diabetes, hypertension, etc., were excluded from PROTECT, because the overarching objective was to obtain a population where we could investigate the role of environmental contaminants specifically in the etiology of preterm birth. Thus, we excluded some of these known risk factors for preterm in order to obtain a population where we would have more statistical power for assessing associations with environmental factors. The rate we observed is similar to that in the US overall (9.57% in 2014) and among all women who identify as Puerto Rican residing in the continental US (11% in 2015) [14]. In regard to birth weight, our study observed similar rates of low birth weight (9.5%) compared to what is observed in Puerto Ricans in the continental US (9.4% in 2015) [14]. Finally, in regard to C-section rates, we had substantially higher rates in our population overall (47%) compared to those seen in the general US (32.2% in 2014) or among Puerto Rican women residing in the continental US (34.2% overall) [14].

Investigation of the risk factors for adverse pregnancy outcomes in Puerto Rico is important, given the high rates reported by the March of Dimes. However, studies of pregnancy in Puerto Rican women residing on the island or elsewhere are sparse. In a study of primarily Puerto Rican women residing in Massachusetts, USA, between 2000–2003, education, parity, pre-pregnancy BMI, weight gain in pregnancy, and history of adverse pregnancy outcomes were associated with risk of SGA [15] but not preterm birth [16]. We did not observe associations with as many factors and SGA; only smoking and season of delivery was associated with an increased OR. This could be due to many differences, such as location, time frame, and sampling criteria, between these two study populations. To our knowledge, this is the first study to examine demographic and pregnancy factors related to adverse birth outcomes in a population residing in Puerto Rico.

Some of the risk factors we identified in our study are consistent with those observed in the general US population. For preterm birth, we observed increased risk in association with lower socioeconomic status (as assessed by household income, education level, and employment status) which is a well-known risk factor [9]. However, we did not observe associations with maternal age, being single, or with tobacco use in pregnancy. These differences may have been due to low numbers in the extremes for these variables. For SGA, we observed a clear increased risk in association with tobacco use in pregnancy which is one of the best-established risk factors for this disease [17]. For LGA, we observed increased risk in association with higher maternal age, but only modest associations with parity and pre-pregnancy BMI which have been previously noted [18]. We might speculate that the latter difference may be due to the fact that women with diabetes were excluded from this study population, and thus women with high BMI may be metabolically healthier than in other studies.

The PROTECT study was designed to investigate factors influencing the notably higher preterm birth rates that have been observed in Puerto Rico compared to the rest of the US. The results from this analysis do little to explain the higher rates of preterm birth that have been observed on the island of Puerto Rico. The most notable difference in this population is poverty. In 2015 46.1% of the Puerto Rican population fell below the US poverty level—the lowest of all US states and territories—as compared to 14.7% of the overall US population [19]. Mississippi, which also experiences among the highest rates of preterm birth in the US, falls second from bottom, with 22% of the population having a household income below the US poverty level [19]. This highlights an important public health disparity that needs to be addressed.

Furthermore, an important area of future investigation will be to understand which risk factors are driving the economic differences in adverse pregnancy outcomes in the Puerto Rican population. All of the women in the present study had access to health care, yet those with lower income or education level had higher risk of delivering preterm. We hypothesize that environmental exposures may be responsible for some of the socioeconomic differences observed in association with preterm birth. We have noted, for example, higher levels of some urinary phthalate metabolites and pesticides in women with lower income or education levels in pilot studies from this population [20, 21]. We plan to investigate socioeconomic disparities in these and other exposures in this study population and determine their role in the etiology of preterm birth and other adverse pregnancy outcomes in future work.

Our present analysis had a number of limitations. It should be noted that the PROTECT cohort has recruited about half of its final anticipated sample size. Thus, addressing questions about more rare pregnancy outcomes (e.g., miscarriage, preeclampsia) was not possible. Investigating factors associated with these endpoints will be a priority when data collection is complete, especially because of the relatively high rates of these outcomes observed in our study population. Additionally, because of our eligibility criteria, the generalizability of our results is restricted to women without diabetes.

## Conclusions

In summary, in a large prospective study of pregnancy in Puerto Rico, we examined for the first time demographic, behavioral, and pregnancy-related risk factors for adverse birth outcomes including preterm and postterm birth as well as small- and large- for gestational age. The most notable findings were associations between low income and education level in relation to preterm birth. Environmental factors including chemical exposures may play an important in the etiology of adverse pregnancy outcomes but have yet to be explored in Puerto Rico. As recruitment into the PROTECT cohort study continues, this analysis will serve as an important reference for understanding of risk and contributing factors to adverse pregnancy outcomes prior to the spread of Zika to the island and the catastrophe of Hurricanes Irma and Maria.

## Supporting information

S1 File Initial screening and first visit questionnaires from the PROTECT project in English and Spanish. (PDF)

S2 File Supporting tables for Demographic risk factors for adverse birth outcomes in Puerto Rico in the PROTECT cohort. Table A. American College of Obstetricians and Gynecologists (ACOG) guidelines for redating gestational age based on ultrasonography. Adapted from ACOG Committee opinion Number 700, May 2017. Table B. Odds ratios (OR) and 95% confidence intervals (CI) for preterm birth in association with demographic and pregnancy characteristics in the PROTECT cohort 2011–2017. Table C. Odds ratios (OR) and 95% confidence intervals (CI) for postterm birth in association with demographic and pregnancy characteristics in the PROTECT cohort 2011–2017. Table D. Adjusted^1^ odds ratios (OR) and 95% confidence intervals (CI) for preterm birth in association with demographic and pregnancy characteristics in the PROTECT cohort 2011–2017. Table E. Adjusted odds ratios (aOR) and 95% confidence intervals (CI) for postterm birth in association with demographic and pregnancy characteristics in the PROTECT cohort 2011–2017. Table F. Odds ratios (OR) and 95% confidence intervals (CI) for small for gestational age (SGA) birth compared to appropriate for gestational age birth (AGA) in association with demographic and pregnancy characteristics in the PROTECT cohort 2011–2017. Table G. Odds ratios (OR) and 95% confidence intervals (CI) for large for gestational age (LGA) birth compared to appropriate for gestational age birth (AGA) in association with demographic and pregnancy characteristics in the PROTECT cohort 2011–2017. Table H. Adjusted^1^ odds ratios (OR) and 95% confidence intervals (CI) for small for gestational age (SGA) birth compared to appropriate for gestational age birth (AGA) in association with demographic and pregnancy characteristics in the PROTECT cohort 2011–2017. Table I. Adjusted^1^ odds ratios (OR) and 95% confidence intervals (CI) for large for gestational age (LGA) birth compared to appropriate for gestational age birth (AGA) in association with demographic and pregnancy characteristics in the PROTECT cohort 2011–2017.
(DOCX)
