# Supplementary material for: Demographic risk factors for adverse birth outcomes in Puerto Rico in the PROTECT cohort
Source: PLoS One. 2019 Jun 13;14(6):e0217770. doi: 10.1371/journal.pone.0217770 (PMC6564423; doi:10.1371/journal.pone.0217770)
Supplement: S1 File — (PDF) [file pone.0217770.s001.pdf]

SITE ID: _____<br>Sj: San Juan UMC<br>Ar: Arecibo<br>Ma: Manati<br>STUDY ID: ____________<br>VISIT ID: V0	**PRoTECT**<br>(Puerto Rico Testsite for Exploring Contamination Threats)<br>**Screening Form (~14 WEEKS)**	START TIME:<br>Time __ __ Hr. __ __ Min.<br>DATE:<br>__ __ / __ __ / __ __ __ __<br>MM DD YYYY<br>INTERVIEWER:<br>______________________

Introduction:

Good morning (evening), my name is __(Interviewer)____. We want to invite you to participate in a research study. The project investigators, from the University of Puerto Rico, University of Michigan, and Northeastern University, want to understand the mechanisms by which the environment may contribute to the health of the fetus and the duration of the pregnancy.

Your participation is voluntary and won't cost you anything. Participation will include an incentive of up to $50 in debit card to be used as you prefer. This study will involve signing a letter of consent and answering a few questions on the days of your visits in order to know your socio-demographic information, food consumption and daily activity patterns which may be related to exposure to environmental pollutants. We will also ask that you provide a sample of blood, urine, hair, and toenails to measure a variety of indicators. At birth, we would like to take a sample of your baby's blood from the umbilical cord. We will also collect DNA samples from you and your baby's blood with the goal of studying genes that are important in the response to environmental exposures.

Are you interested in learning more about this project and would you consider participating?

Yes → (Continue Questionnaire) No →Thank participant for her time

Would it be possible to know your age and city of residence (If not, finish interview)

If Yes→ Age ______ City ______________________________

Note: Skip to Section D and select the reason for not participating

---

1. What is your full name?

______________________ ______________________ ______________________ ______________________
Name Last Name- Paternal Last Name-Maternal

2. What is your current age? |___|___| Years (If Age is less than 18 and above 40, participant is excluded)

3. Where are you planning to deliver your baby?

□ Hospital Universitario, San Juan (1)
□ Manati Medical Center, Manatí (2)
□ Hospital Cayetano Coll y Toste, Arecibo (3)
□ Other (specify) ________________________________ (97)

4. In which municipality do you live? __________________________

List of municipalities that are eligible: Aguadilla, Arecibo, Barceloneta, Camuy, Ciales, Corozal, Dorado, Florida, Hatillo, Isabela, Lares, Manatí, Moca, Morovis, Orocovis, Quebradillas, San Sebastián, Utuado, Vega Alta, Vega Baja (Note: If participant does not reside in any of these municipalities, finish the interview and thank her for her time.)

SECTION A:

1. Do you remember the date of your last menstruation? ☐ Yes (1) ☐ No (0)

If YES, please enter the date ___________________________ (mm/dd/yy)

Interviewer, calculate gestational age estimated on the basis of last menstrual period (LMP) (Don't Ask)

__________________ (Estimated Gestational Age in weeks)

__________________ (Expected due date)

2. Have you received an ultrasound to estimate your due date? ☐ Yes (1) ☐ No (0)

If YES, please enter the date the ultrasound exam was performed _________________ (mm/dd/yy)

What is the estimated gestational age by ultrasound? ____________ weeks

Interviewer: Is ultrasound-estimated gestational age <20 weeks (Don't Ask)?

☐ Yes (1) ☐ No (0) ☐Ultrasound not performed

SECTION B:

1. Had you been taking birth control pills in the three months prior to becoming pregnant? ☐ Yes (1) ☐ No (0)
2. Did you use *in vitro* fertilization (IVF) to become pregnant for this pregnancy? ☐ Yes (1) ☐ No (0)
3. Did your doctor inform you that you are carrying more than one baby? ☐ Yes (1) ☐ No (0)
4. Have you had any medical or obstetric difficulties during this pregnancy? ☐ Yes (1) ☐ No (0)

Abortion Threat	☐ Yes (1) ☐ No (0)
Vaginal Bleeding or Strong Abdominal pain (no diagnose)	☐ Yes (1) ☐ No (0)
Diabetes before pregnancy	☐ Yes (1) ☐ No (0)
Cardiovascular disease before pregnancy	☐ Yes (1) ☐ No (0)
High blood pressure	☐ Yes (1) ☐ No (0)
Kidney Disease	☐ Yes (1) ☐ No (0)
Neuropathy	☐ Yes (1) ☐ No (0)
Liver disease before pregnancy	☐ Yes (1) ☐ No (0)
Surgery during this pregnancy	☐ Yes (1) ☐ No (0)

(**Note to interviewer** – participant must answer "YES" to one question in section A, and have less than 20 weeks of gestational age. If the participant answered "NO" to any question in section A, "YES" to any in Section B or has more than 20 weeks estimated gestational age, finish screening and thank participant. Also, participant must plan to deliver at one of the three hospitals listed and live in the karst region to be eligible.)

SECTION C

1. Is the participant eligible to participate in this study? ☐ Yes (1) ☐ No (0)

(Answered "YES" to one of the questions in section A, and "NO" to all in section B)

If NO, thank participant for her time and end screening

SECTION D

1. Is the participant willing to participate in this study?

☐ Yes (1) Give the participant the consent form for signing, give time to read, and attend any questions

☐ No (0)

If NO, indicate the reason and thank participant for her time and end screening:

a. ☐ No time (1)
b. ☐ Live far away (2)
c. ☐ Do not want to give samples (3)
d. ☐ Other (specify) ______________________________ (4)

Contact Information:

1. Current Residential Address:

____
____
____
____

References (Ex. Cross-streets or places of interest)

____

2. Current Work Address:

____
____
____
____

3. Telephones:

Home _________________________ Mobile ____________________________

Work __________________________ Other ____________________________

If participant is eligible and signed the consent form , assign participant ID

Assign ID number ____________________

(Range: Sj/Ar/Ma-001 through Sj/Ar/Ma-900 for 900)

Site ID is **Sj** for San Juan, **Ar** for Arecibo, **Ma** for Manati

If not eligible, assign non-participant ID (range NP0001 – NPXXXX)

Non-Participant ID: _________________________

Time and date of the next appointment (visit): ______________________________________

Time at the end of interview: _______________ (Hrs, Min)

Código del Lugar: _____

Sj: San Juan UMC
Ar: Arecibo
Ma: Manatí

ID: ___________

Visita #: V0

PRoTECT

(Puerto Rico como Lugar para Explorar Amenazas por Contaminación Ambiental)

Cuestionario de Cernimiento (~14 SEMANAS)

Hora al Comienzo:

Hora: __ __ Hr. __ __ Min.

Fecha:

__ __ / __ __ / __ __ __ __
MM DD YYYY

Entrevistador:
____

Introducción:

Buenos días (tardes), mi nombre es __(Entrevistador)_______.Queremos invitarla a participar en un estudio de investigación. Los investigadores del proyecto de la Universidad de Michigan, Universidad de Puerto Rico y la Universidad de Northeastern, quieren entender los mecanismos por los cuales la contaminación ambiental puede contribuir a la salud del feto y la duración del embarazo.

Su participación es voluntaria y no le generará ningún costo. La participación incluye un incentivo de hasta $50 dólares en tarjetas de débito para ser utilizada a su preferencia. Dicho estudio consistirá en firmar una carta de consentimiento y contestar unas preguntas que se le harán en los días de su visita para saber su información socio-demográfica, consumo de alimentos y actividades diarias relacionadas a exposición a contaminantes ambientales. También le pediremos una muestra de sangre, orina, pelo y uñas de los pies para medir varios biomarcadores. Durante el nacimiento, nos gustaría tomar una muestra de sangre del cordón umbilical del recién nacido, así como también una muestra de placenta. También se le colectará una muestra de ADN a usted y a su bebé con el propósito de estudiar genes que son importantes en la respuesta a exposiciones ambientales.

¿Le interesaría saber más sobre este estudio y consideraría participar?

Sí → continúe con el cuestionario. No → Gracias por su tiempo.

¿Sería posible saber su edad y municipio de residencia?

Sí →, obtener edad y municipio.

Edad _____ Municipio _______________

No →, Terminar la entrevista. Pasar a la Sección D y seleccionar la razón por la que no desea participar.

1. Nombre ____

2. Edad actual _______

(Si tiene menos de 18 años o es mayor de 40 años terminar la entrevista. Informarle que por su edad no es elegible para el estudio. Gracias por su tiempo.)

3. ¿En qué Hospital planifica dar a luz a su bebé?

a. ☐ Hospital Universitario, San Juan
b. ☐ Manatí Medical Center, Manatí
c. ☐ Hospital Cayetano Coll y Toste, Arecibo
d. ☐ Otro (Especifique) ______________________

4. ¿En qué municipio vive? ______________________

Lista de municipios elegibles: Aguadilla, Arecibo, Barceloneta, Camuy, Ciales, Corozal, Dorado, Florida, Hatillo, Isabela, Lares, Manatí, Moca, Morovis, Orocovis, Quebradillas, San Sebastián, Utuado, Vega Alta, Vega Baja (**Nota**: Si no reside en uno de los municipios participantes, terminar la entrevista)

Sección A:

1. ¿Se acuerda usted de la fecha de su última menstruación? (1) ☐ Sí (0) ☐No
   Si contesta SÍ, por favor ingrese la fecha ______________________ (mm/dd/yy)

Entrevistador: Calcule la edad gestacional estimada en base a la fecha de la última menstruación

__________________ (Edad Gestacional Estimada en semanas)

__________________ (Fecha Esperada de Parto)

2. ¿Ha recibido un ultrasonido para estimar la edad gestacional? (1) ☐ Sí (0) ☐ No

   Si contesta SÍ, por favor ingrese la fecha en que se realizó el ultrasonido __________ (mm/dd/yy)

   ¿Cuál fue la edad gestacional estimada por este ultrasonido? __________ semanas

Entrevistador: ¿Es la fecha estimada por ultrasonido < 20 semanas?

(1) ☐ Sí (0) ☐ No (2) ☐ NO SE HIZO ULTRASONIDO

Sección B:

1. ¿Ha estado tomando pastillas anticonceptivas en los tres meses antes de quedar embarazada?
   (1) ☐ Sí (0) ☐ No
2. ¿Utilizó fertilización in-vitro para embarazarse en este embarazo?
   (1) ☐ Sí (0) ☐ No
3. ¿Su doctor le ha informado que carga más de un bebé? (1) ☐ Sí (0) ☐ No
4. ¿Ha tenido alguna de las siguientes dificultades médicas u obstétricas durante este embarazo?
   (1) ☐ Sí (0) ☐ No

Amenaza de aborto	(1) □ Sí	(0) □ No
Sangrado vaginal o dolor abdominal fuerte (sin diagnosticar)	(1) □ Sí	(0) □ No
Diabetes antes del embarazo	(1) □ Sí	(0) □ No
Enfermedad cardiaca antes del embarazo	(1) □ Sí	(0) □ No
Presión arterial elevada	(1) □ Sí	(0) □ No
Nefropatía	(1) □ Sí	(0) □ No
Neuropatía	(1) □ Sí	(0) □ No
Enfermedad del hígado antes del embarazo	(1) □ Sí	(0) □ No
Cirugía durante este embarazo	(1) □ Sí	(0) □ No

(**Nota al entrevistador** – la participante debe de contestar "SÍ" a por lo menos una de las preguntas en la sección (A), y tener menos de 20 semanas de edad gestacional estimada. Si contesta "NO" en las 2 preguntas de la Sección (A), "SÍ" en algunas de las preguntas de la sección (B), o tiene más de 20 semanas de gestación, finalice el tamizaje y agradezca a la participante. También, la participante debe dar a luz en uno de los tres hospitales listados en la pregunta #3 y vivir en el área del karso para ser elegible.

Sección C:

1. ¿Es la participante elegible para participar en el estudio? (Ha contestado "SÍ" a por lo menos una de las preguntas en la Sección (A) y "NO" en todas las preguntas en la sección (B)

   (1) □ Sí (0) □No

Si la respuesta es "NO", agradezca a la participante por su tiempo y finalice el tamizaje.

Sección D:

1. ¿Está dispuesta a participar en este estudio?

   (1) □ Sí → Proceder a entregarle la hoja de consentimiento y ofrecer la oportunidad de leerla y contestar preguntas y complete el proceso con la firma de la participante.
   (0) □No

Si la respuesta es "NO", indique la razón y agradezca a la participante por su tiempo y finalice la entrevista.

a. □ No tiene tiempo
b. □ Vive muy lejos
c. □ No quiere proveer muestras
d. □ Otras (Especifique) ______________________________

Si es elegible y firmó la hoja de consentimiento, asignar número de participante

Información de Contacto

1. Dirección de Residencia Actual

____
____
____
____

Referencias (Ej. calles cercanas, calle transversal o lugares de interés)

____

2. Dirección del Trabajo Actual:

____
____
____
____

3. Teléfonos:

Casa ________________________ Celular ___________________________

Trabajo ______________________ Otro _________________________

Asignar Número de Identificación___________

(Rango: Sj/Ar/Ma 001 hasta Sj/Ar/Ma 900 para un total de 900 participantes)
ID de Sitio: **Sj** para San Juan, **Ar** para Arecibo, **Ma** para Manatí

Identificar el lugar donde se reclutó la participante _____

1. Manatí Medical Center
2. Ciales Primary Health Care Services
3. MOMS
4. Doctors Center, Manatí
5. Morovis Health Center
6. Centro de Salud de Lares
7. Centro de Salud de Lares, Quebradillas
8. Cayetano Call y Toste, Arecibo
9. Camuy Health Services
10. Otro (Especifique) ____________________________

<u>Si no es elegible, asignar ID-No Participante (Rango NP 001 – NP XXX)</u>

ID No Participante _________________

Hora y fecha de la próxima cita (visita): _________________________

Hora al final de la entrevista: ________________________

SITE ID: ____<br><br>Sj: San Juan UMC<br>Ar: Arecibo<br>Ma: Manati<br><br>STUDY ID: ___ ___ ___ ___<br><br>VISIT ID: V1	**PRoTECT**<br>(Puerto Rico Testsite for Exploring Contamination Threats)<br><br>**First Visit (~20 WEEKS) Questionnaire**<br><br>Demographic Information, Employment Status<br>Residential History, etc.…	START TIME:<br><br>Time __ __ Hr. __ __ Min.<br><br>DATE:<br>__ __ / __ __ / __ __ __ __<br>MM DD YYYY<br><br>INTERVIEWER:<br>______________________

Introduction:

Good morning (afternoon/evening). Thank you for agreeing to meet with us. I would like to ask you some questions about your background: Where you have lived, the jobs you have had, your medical history and your eating and other personal habits. Some of the questions are sensitive in nature, so you don't have to answer any that you choose not to. We are trying to learn how these things may affect health. We would appreciate your help in answering these questions as best you can. But, you don't have to answer any questions that you'd rather not answer. Everything you tell me will be kept private and will not be shared with anyone outside the study.

During the interview, please feel free to ask me to slow down or repeat any question that is unclear. Before I begin, I want to remind you that everything you tell me will be kept private.

---

1. What is your full name?

____________________ ____________________ ____________________ ____________________
Name Last Name- Paternal Last Name-Maternal

2. When were you born? (month/day/year) ____/____ /____

3. What is your estimated due date?

|___|___| / |___|___| / |___|___|
MM DD YY

REFUSE.. 98
DON'T KNOW 99

Gestational Age:

a. Last Menstrual Period (LMP) Date: __ __/__ __/__ __ (mm /dd / yy)

b. Gestational Age by Ultrasound __________

4. Have you changed residential address since the last visit?
Yes 1
No.. ….. 0 (SKIP TO QUESTION 6)

5. What is your Current Address?

Street___________________________________ No._________

Urb./Bldg. ____ Apt. # ___________

City ______________________ Zip Code _________

Reference: (cross street or landmark):

____

6. Place of Origin (city and corresponding state)

A. Where were you born? ___________________________
B. Where was your mother born? _____________________
C. Where was your father born? ______________________

DEMOGRAPHIC INFORMATION

7. Marital Status

Single	_____ (1)
Married	_____ (2)
Divorced	_____ (3)
Widow	_____ (4)
Living together, not married	_____ (5)
Refused	_____ (98)
Don't Know	_____ (99)

8. Do you consider yourself to be Hispanic, or Latina?

NO ....(0) (SKIP TO QUESTION 10)
YES ....(1)
REFUSED ....(98)
DON'T KNOW ....(99)

9. Which of the following represents your Hispanic origin or ancestry?

PUERTO RICAN 1
CUBAN/CUBAN AMERICAN 2
DOMINICAN REPUBLIC 3
MEXICAN 4
MEXICAN AMERICAN 5
CENTRAL OR SOUTH AMERICAN 6
OTHER (SPECIFY):____ 97
REFUSED 98
DON'T KNOW 99

10. What race do you consider yourself to be? You may select one or more. (No-0, Yes-1, Refused-98, Don't Know-99)

a. White, ..._____
b. Black or African American, ..._____
c. American Indian or Alaska Native, ..._____
d. Asian, or ...._____
e. Native Hawaiian or Other Pacific Islander? ...._____
f. Mixed Race .... _____
g. SOME OTHER RACE? (SPECIFY): _________________________

11. What is the **highest** degree or level of school that you have completed?

NO SCHOOL 01

ELEMENTARY
NURSERY SCHOOL TO 4TH GRADE 02
5TH-6TH GRADE 03
7TH-8TH GRADE 04

HIGH SCHOOL
9TH GRADE 05

10TH GRADE 06
11TH GRADE 07
12TH GRADE (NO DIPLOMA) 08
HIGH SCHOOL DIPLOMA 09
GED OR EQUIVALENT 10

COLLEGE
SOME COLLEGE CREDITS, BUT LESS THAN 1 YEAR 11
1 OR MORE YEARS OF COLLEGE, BUT NO DEGREE 12
ASSOCIATE DEGREE: OCCUPATIONAL, TECHNICAL, OR VOCATIONAL PROGRAM 13
ASSOCIATE DEGREE: ACADEMIC PROGRAM 14
BACHELOR'S DEGREE (e.g., BA, BS) 15

GRADUATE
MASTER'S DEGREE (e.g., MA, MS, MSW, MEng, MBA) 16
PROFESSIONAL SCHOOL DEGREE (e.g., MD, DDS, DVM, JD) 17
DOCTORAL DEGREE (e.g., Ph.D., Ed.D.) 18

REFUSED 98
DON'T KNOW 99

Now I am going to ask you some questions about the baby's heritage on the father's side.

12. Do you consider ***your baby's father*** to be Hispanic, or Latino?

NO .... 0 (SKIP TO QUESTION 14)
YES 1
REFUSED 98
DON'T KNOW 99

13. Which of the following represents ***your baby's father's*** Hispanic origin or ancestry?

PUERTO RICAN 1
CUBAN/CUBAN AMERICAN 2
DOMINICAN (REPUBLIC) 3
MEXICAN 4
MEXICAN AMERICAN 5
CENTRAL OR SOUTH AMERICAN 6
OTHER (SPECIFY):____________________ 97
REFUSED 98
DON'T KNOW 99

14. What race do you consider ***your baby's father*** to be? You may select one or more. (No-0, Yes-1, Refused-98, Don't Know-99)

a. White, ..._____
b. Black or African American, ..._____
c. American Indian or Alaska Native, ..._____
d. Asian, or ...._____
e. Native Hawaiian or Other Pacific Islander? ...._____
f. Mixed Race ...._____
g. SOME OTHER RACE? (SPECIFY): ____________________

15. What is the **highest** degree or level of school that your baby's father has completed?

NO SCHOOL 01

ELEMENTARY
NURSERY SCHOOL TO 4TH GRADE 02
5TH-6TH GRADE 03
7TH-8TH GRADE 04

HIGH SCHOOL
9TH GRADE 05
10TH GRADE 06
11TH GRADE 07
12TH GRADE (NO DIPLOMA) 08
HIGH SCHOOL DIPLOMA 09
GED OR EQUIVALENT 10

COLLEGE
SOME COLLEGE CREDITS, BUT LESS THAN 1 YEAR 11
1 OR MORE YEARS OF COLLEGE, BUT NO DEGREE 12
ASSOCIATE DEGREE: OCCUPATIONAL, TECHNICAL, OR VOCATIONAL PROGRAM 13
ASSOCIATE DEGREE: ACADEMIC PROGRAM 14
BACHELOR'S DEGREE (e.g., BA, BS) 15

GRADUATE
MASTER'S DEGREE (e.g., MA, MS, MSW, MEng, MBA) 16
PROFESSIONAL SCHOOL DEGREE (e.g., MD, DDS, DVM, JD) 17
DOCTORAL DEGREE (e.g., Ph.D., Ed.D.) 18

REFUSED 98
DON'T KNOW 99

EMPLOYMENT STATUS

Now I would like to ask some questions about any schoolwork, jobs, volunteer work, and hobbies that you have done recently. ***Please only include activities that you do or have done for at least four hours per week.***

16. Are you currently a full- or part-time student? This includes vocational or technical schooling that may not be done in a classroom.

NO, NOT A STUDENT .... 0 (SKIP TO QUESTION 20)
YES, FULL-TIME STUDENT 1
YES, PART-TIME STUDENT 2
REFUSED .... 98 (SKIP TO QUESTION 20)
DON'T KNOW 99

17. What type or types of school are you currently attending? You may select more than one. (No-0, Yes-1, Refused-98, Don't Know-99)

a. HIGH SCHOOL .... _____
b. TECHNICAL SCHOOL .... _____
c. COLLEGE OR UNIVERSITY .... _____
d. GRADUATE SCHOOL .... _____
e. PROFESSIONAL SCHOOL (E.G., MEDICAL, LAW, DENTAL) .... _____
f. OTHER (SPECIFY): ____

18. What best describes the place where you typically go to school? You may select one or more. (No-0, Yes-1, Refused-98, Don't Know-99)

a. CLASSROOM .... _____
b. RESIDENCE, E.G. YOUR HOME OR SOMEONE ELSE'S HOME ..... _____
c. LABORATORY .... _____
d. GARAGE OR SHOP .... _____
e. MOTOR VEHICLE .... _____
f. SOME OTHER LOCATION (SPECIFY): ______________________

19. (Please tell me the address where you **actually** attend school most often.)

Street___________________________________ No._________

Urb./Bldg. ____ Apt. # ___________

City ______________________ Zip Code _________

Reference: (cross street or landmark) : ____

REFUSED 98
DON'T KNOW 99

20. Now I would like to ask you about jobs you have had recently.

Since you became pregnant,

		NUMBER	RF	DK
19a.	How many full-time jobs have you had?............................	|___|___|	98	99
19b.	How many part-time jobs have you had?	|___|___|	98	99
19c.	How many volunteer jobs have you had (fire department, humane society, etc.)?	|___|___|	98	99

21. Are you currently employed?

NO .... 0 (SKIP TO QUESTION 35)
YES 1
REFUSED .... 98 (SKIP TO QUESTION 35)
DON'T KNOW .... 99 (SKIP TO QUESTION 35)

22. For this job, what is your job title or occupation?

____
JOB TITLE

REFUSED 98
DON'T KNOW 99

23. For this job, who is your employer?

____
EMPLOYER

REFUSED 98
DON'T KNOW 99

24. What types of activities do you do most often at this job? For example, teach classes, work on the computer, keep account books, file, photocopy, answer phone, wait tables, help customers, do lab work, or carpentry? PROBE: Anything else?

____
ACTIVITY

REFUSED 98
DON'T KNOW 99

25. In what kind of business or industry is this job? That is, what does this company make or do?

____
INDUSTRY

REFUSED 98
DON'T KNOW 99

26. On average, how many hours a week do you usually work at this job?

|___|___|___|
NUMBER OF HOURS

REFUSED 98
DON'T KNOW 99

27. What is your work shift?

Day Shift 1
Evening Shift 2
Night Shift 3
Rotating Shifts 4
REFUSED 98
DON'T KNOW 99

28. How often do you lift or move heavy objects at this job?

Never 1
Sometimes 2
Often 3
REFUSED 98
DON'T KNOW 99

29. What is the level of physical exertion at this job?

Light 1
Moderate 2
Heavy 3
REFUSED 98
DON'T KNOW 99

30. What is the normal temperature in your workplace?

Normal (68-73°F) room temperature 1
Above room temperature 2
Below room temperature 3
REFUSED 98
DON'T KNOW 99

31. Which of the following best describes the place where you typically work for this job? (Select one)

a. OFFICE AREA	01
b. STORE	02
c. CLASSROOM	03
d. HOTEL OR MOTEL	04
e. RESTAURANT	05
f. RESIDENCE, SUCH AS YOUR HOME OR SOMEONE ELSE'S HOME	06
g. HEALTHCARE FACILITY OR HOSPITAL	07
h. LABORATORY	08
i. FACTORY, PLANT, OR PRODUCTION AREA	09
j. WAREHOUSE	10
k. GARAGE OR SHOP	11
l. SALON	12
m. LOADING DOCK	13
n. CONSTRUCTION SITE	14
o. GROUNDS, YARD, OR GARDEN	15
p. BARNS, FIELD, OR FARMYARDS	16
q. MOTOR VEHICLE	17
r. SOME OTHER LOCATION (SPECIFY):______________________	97
s. REFUSED	98
t. DON'T KNOW	99

32. What is the address where you **actually** work at this job?

HOME	1
VARIES (CONSTRUCTION, LANDSCAPING)	2
HAVE EXACT ADDRESS	3
OTHER (SPECIFY):____	97
REFUSED	98
DON'T KNOW	99

33. Have you changed work address since we last met?

Yes	1
No	0 (SKIP TO QUESTION 35)

34. Please tell me the address where you **actually** work at this job.

Street___________________________________ No._________

Urb./Bldg. ____ Apt. # ___________

City ______________________ Zip Code _________

Reference: (cross street or landmark) ____

REFUSED	98
DON'T KNOW	99

INCOME

35. Of these income groups, which category best represents your household/family income last year? Remember, a family is a group of two or more people who live together and who are related by birth, marriage, or adoption.

Less than $4,999	01

$5,000-$9,999	02
$10,000-$19,999	03
$20,000-$29,999	04
$30,000-$39,999	05
$40,000-$49,999	06
$50,000-$74,999	07
$75,000-$99,999	08
$100,000-$199,000	09
$200,000 or more	10
REFUSED	98
DON'T KNOW	99

36. Are there any other family members, not living in this household, who are also supported by this income?

No	0 (SKIP TO QUESTION 38)
Yes	1
REFUSED	98 (SKIP TO QUESTION 38)
DON'T KNOW	99 (SKIP TO QUESTION 38)

37. How many other family members, not living in this household, are supported by this income?

|___|___|
NUMBER

REFUSED	98
DON'T KNOW	99

TOBACCO HABITS

Now I'd like to ask you some questions about tobacco use.

38. Do you currently smoke?

NO	_____	(0)
YES	_____	(1)
REFUSED	_____	(98)
DON'T KNOW	_____	(99)

39. In your lifetime, have you smoked as many as 100 cigarettes?

NO	_____	(0) (SKIP TO QUESTION 46)
YES	_____	(1)
REFUSED	_____	(98)
DON'T KNOW	_____	(99)

40. Was there ever at time that you smoked at least 1 cigarette a day for a month or longer?

NO	_____	(0) (SKIP TO QUESTION 46)
YES	_____	(1)
REFUSED	_____	(98)
DON'T KNOW	_____	(99)

41. For about how many years total would you say that you smoked at least 1 cigarette per day?

___ ___ years smoked (Code Refused as 98, Don't Know as 99)

42. During the time you smoked at least 1 cigarette a day, about how many cigarettes a day on average?

___ ___ cigarettes/day on average (Code Refused as 98, Don't Know as 99)

43. When was your last cigarette?

Today	_____	(0)
In the past week	_____	(1)
More than a week ago	_____	(2)
More than a month ago	_____	(3)
Before pregnancy	_____	(4)
Refused	_____	(98)
Don't Know	_____	(99)

44. Did you ever quit smoking for 6 months or longer?

NO	_____	(0) (SKIP TO QUESTION 44)
YES	_____	(1)
REFUSED	_____	(98) (SKIP TO QUESTION 44)
DON'T KNOW	_____	(99)

44a. Did you quit because of your pregnancy?

NO	_____	(0)
YES	_____	(1)
REFUSED	_____	(98)
DON'T KNOW	_____	(99)

45. If you stopped smoking cigarettes and then started smoking again, for how many years did you quit?

___ ___ years quit (Code Refused as 98, Don't Know as 99)

46. Does anyone else in your household smoke on a daily basis?

NO	_____	(0) (SKIP TO QUESTION 48)
YES	_____	(1)
REFUSED	_____	(98)
DON'T KNOW	_____	(99)

46a. Who smokes in your household, and how many cigarettes do they smoke per day on average?
(No-0, Yes-1, Refused-98, Don't Know-99)

a. Father (grandparent of baby)	____	_____ cigarettes/day on average
b. Mother (grandparent of baby)	____	_____ cigarettes/day on average
c. Spouse	____	_____ cigarettes/day on average
d. Sibling	____	_____ cigarettes/day on average
e. Other	____	_____ cigarettes/day on average

47. Since you became pregnant, how many hours per day, on average, have you been around someone else who is smoking, close enough for you to smell the smoke?

____ ____ hrs/day (Code Refused as 98, Don't Know as 99)

ALCOHOL USE

The next three questions are about alcohol drinking. When I ask about the number of drinks, I mean that a 12oz bottle of beer, a 4oz glass of wine, and a shot of liquor are each considered to be equal to one drink. Also, the questions ask about usual drinking patterns, rather than occasional (at parties or celebrations, for instance).

48. When did you last have an alcoholic drink?

Do not drink alcohol	_____	(0) (SKIP TO QUESTION 51)
Today	_____	(1)
Yesterday	_____	(2)
Sometime last week	_____	(3)

More than a week ago	_____	(4)
More than a month ago	_____	(5)
Before pregnancy	_____	(6)
Refused	_____	(98)
Don't Know	_____	(99)

49. During the past year, about how many **days in a week** did you usually drink alcohol?

None	_____	(0) (SKIP TO QUESTION 51)
1 to 2 days	_____	(1)
3 or more days	_____	(2)
Refused	_____	(98)
Don't Know	_____	(99)

50. During the past year, each time you had alcohol, about how **many drinks** did you usually have?

None	_____	(0)
1 to 2 drinks	_____	(1)
3 to 5 drinks	_____	(2)
6 or more drinks	_____	(3)
Refused	_____	(98)
Don't Know	_____	(99)

DRUG USE

Next I am going to ask you some questions about drugs that you might take. I want to remind you that everything you tell me will be kept absolutely private and will not be shared with anyone.

51. Are you currently smoking **marijuana**?

NO	_____	(0)
YES	_____	(1)
REFUSED	_____	(98)
DON'T KNOW	_____	(99)

52. Are you currently using **other illicit or street drugs**, including drugs that you smoke or inject?

NO	_____	(0) (SKIP TO QUESTION 53)
YES	_____	(1)
REFUSED	_____	(98) (SKIP TO QUESTION 53)
DON'T KNOW	_____	(99)

52a. If YES, what drugs are they? (Code Refused as 98, Don't Know as 99)

I. ______________________
II. ______________________
III. ______________________
IV. ______________________

WATER USE

Now I'd like to ask you some questions about your use of water

53. What is the primary source of water in your home for drinking?

PR Acueduct and Sewer Authority (PRASA or AAA)	______	(1)
Private Well	______	(2)
Non-PRASA Community Well	______	(3)
Non-PRASA Source-Surface Waters	______	(4)
Cistern (Water Tank)	______	(5)
Rainwater Collection	______	(6)
Bottled/Delivered Water	______	(7)
Refused	______	(98)

Don't Know ______ (99)

54. What is the primary source of water in your home for cooking?

PR Acueduct and Sewer Authority (PRASA or AAA) ______ (1)
Private Well ______ (2)
Non-PRASA Community Well ______ (3)
Non-PRASA Source-Surface Waters ______ (4)
Cistern (Water Tank) ______ (5)
Rainwater Collection ______ (6)
Bottled/Delivered Water ______ (7)
Refused ______ (98)
Don't Know ______ (99)

55. Do you store the water used for drinking and cooking purposes at your residence (i.e. use a cistern)?

NO _____ (0) (SKIP TO QUESTION 56)
YES _____ (1)
REFUSED _____ (98) (SKIP TO QUESTION 56)
DON'T KNOW _____ (99) (SKIP TO QUESTION 56)

55a. If YES, what is the storage container made of?

Plastic _____ (1)
Metal _____ (2)
Other _____ (97)
Refused _____ (98)
Don't Know _____ (99)

56. Do you treat your drinking water at home?

NO _____ (0) (SKIP TO QUESTION 57)
YES _____ (1)
REFUSED _____ (98) (SKIP TO QUESTION 57)
DON'T KNOW _____ (99) (SKIP TO QUESTION 57)

56a. If YES, what kind of treatment?

Cartridge filter _____ (1)
Reverse Osmosis _____ (2)
Boiling _____ (3)
Water Softener _____ (4)
Other _____ (97)
Refused _____ (98)
Don't Know _____ (99)

57. How often do you drink filtered/treated water?

Almost never or never _____ (1)
About ¼ of the time _____ (2)
About ½ of the time _____ (3)
About ¾ of the time _____ (4)
Almost always or always _____ (5)
Refused _____ (98)
Don't Know _____ (99)

PERSONAL MEDICAL HISTORY

I am going to now ask you some questions about your current health

Do you have any of the current symptoms?
Please indicate (No-0, Yes-1, Refused- 98, Don't Know - 99)

58. Headache ______ 58a: Episodes per day: [1-2] ___(1), [2-4] ___(2), [> 4] ___(3)

59. Dizziness ______ 59a. Episodes per day: [1-2] ___(1), [2-4] ___(2), [> 4] ___(3)

60. Nausea ______ 60a. Episodes per day: [1-2] ___(1), [2-4] ___(2), [> 4] ___(3)

61. Vomiting Daily ______ 61a. Episodes per day: [1-2] ___(1), [2-4] ___(2), [> 4] ___(3)

62. Hear a Buzz ______ 62a. Episodes per day: [1-2] ___(1), [2-4] ___(2), [> 4] ___(3)

63. See lights (Stars) ______ 63a. Episodes per day: [1-2] ___(1), [2-4] ___(2), [> 4] ___(3)

64. Fetal Movement (1) Yes _____ (0) No _____

64a. If YES: Is it moving as usual (i.e. time of day, frequency)? (1)Yes ____ (0) No ____

64b. If NO on 62a: Do you feel that the movements have decreased? (1)Yes ____ (0) No ____

65. Contractions (1) Yes _____ (0) No _____

66. If you experience them, how frequent are they?

_______ in one week, __________ in one day, _______ in one hour, ___________in 10 minutes

67. Discharge of liquid/blood/fluid from vagina ______

67a. If YES, what are the characteristics of the vaginal discharge? Select all that apply. (No-0, Yes-1, Refused-98, Don't Know-99)

I. Transparent ______

II. White ______

III. Speckled ______

IV. Fetid ______

V. Yellow ______

VI. Green ______

VII. Coffee ______

VIII. Sparkling blood ______

68. Burning sensation when urinating ______

69. More frequent urination than usual ______

70. Swollen feet ______

71. Currently, would you say your health in general is

Excellent... 1
Very good... 2
Good.. 3
Fair 4
Poor.. 5
REFUSED 98
DON'T KNOW 99

72. How many dental fillings do you have?

|___|___|
#FILLINGS

REFUSED .... 98 (SKIP TO QUESTION 74)
DON'T KNOW .... 99 (SKIP TO QUESTION 74)

73. How many of these fillings are silver in color?

|___|___|
#FILLINGS

REFUSED 98
DON'T KNOW 99

REPRODUCTIVE HISTORY

74. Regarding this pregnancy, were you trying to become pregnant?

Yes 1
No.. 0 (SKIP TO QUESTION 77)
REFUSED 98
DON'T KNOW 99

75. For about how many months were you trying to become pregnant? If 1 month or less, enter 1 and continue to question 77

|___|___|
MONTHS

REFUSED 98
DON'T KNOW 99

76. Were you given any of the following drugs to use to improve your ovulation for **this pregnancy**? Select all that apply (No-0, Yes-1, Refused-98, Don't Know-99).

a. CLOMID ....______
b. GONAL F..______
c. BRAVELLE ....______
d. FOLLISTIM..______
e. REPRONEX ....______
f. PERGONAL ....______
g. PREGNYL ....______
h. PROFASI..______
i. NOVAREL ....______
j. OTHER DRUG (SPECIFY): ___________________________________
k. REFUSED .... ______
l. DON'T KNOW .... ______

77. What was your weight just before you became pregnant?

|___|___|___| OR |___|___|___|
POUNDS (1) KILOGRAMS (2)

REFUSED 98
DON'T KNOW 99

78. How old were you when you had your first menstrual period?

|___|___|
AGE

REFUSED 98
DON'T KNOW 99

79. How many pregnancies have you had? (including this one)

|___|___| pregnancies (Code Refused as 98, Don't Know as 99)

80. How many live-born children have you had? (not including this one)

|___|___| live-born children (Code Refused as 98, Don't Know as 99)

81. How old were you when you first gave birth?

|___|___| yrs (Code Refused as 98, Don't Know as 99)

82. In any of your previous pregnancies have you had a Caesarean section?

NO	_____	(0)
YES	_____	(1)
REFUSED	_____	(98)
DON'T KNOW	_____	(99)

82a. If YES, how many Caesarean sections have you had?

|___|___| Caesarean sections (Code Refused as 98, Don't Know as 99)

82b. How many times have you had a miscarriage (spontaneous abortion)?

|___|___| times (Code Refused as 98, Don't Know as 99)

83. How many children have you had that weighed under 5.5lbs kg at birth or that the doctor told you were low birth weight?

|___|___| children (Code Refused as 98, Don't Know as 99)

84. How many children have you had that were born before the 37th week or that the doctor told you were premature?

|___|___| children (Code Refused as 98, Don't Know as 99)

85. How many children have you had that were stillborn?

|___|___| children (Code Refused as 98, Don't Know as 99)

86. During your previous pregnancies, have you had any of the following complications? (No-0, Yes-1, Refused-98, Don't Know-99)

a. Does Not Apply (First Pregnancy)	______ (SKIP TO QUESTION 87)
b. Bleeding 1st Trimester	______
c. Bleeding 2nd Trimester	______
d. Bleeding 3rd Trimester	______
e. Premature Rupture of the Membrane (PROM)	______
f. Preeclampsia	______
g. Eclampsia	______
h. Heart Disease	______
i. Neuropathy	______
j. Gestational Diabetes	______
k. Anemia	______
l. Vaginal Infections	______
m. Sexually Transmitted Diseases	______

87. Have you had any complications in **this** pregnancy? (No-0, Yes-1, Refused-98, Don't Know-99)

a. Bleeding	______

b. Anemia ______
c. Vaginal Infections ______
d. Urinary tract infection ______
e. Sexually Transmitted Disease ______
f. Premature Rupture of the Membranes (PROM) ______
g. Preeclampsia ______
h. Eclampsia ______
i. Heart Disease ______
j. Neuropathy ______
k. Gestational Diabetes ______

88. How many weeks pregnant were you when you first learned that you were pregnant?

_______ Weeks (Code Refused as 98, Don't Know as 99)

89. When did you start prenatal care under a health professional's supervision?

___ ___/ ___ ___/___ ___ (Code Refused as 98, Don't Know as 99)
Month / Day / Year

DIETARY SUPPLEMENTS

90. In the 3 months before you became pregnant, did you take any prenatal, or multivitamins? *A multivitamin is one pill that contains several different vitamins and minerals, such as One-A-Day or Centrum.*

NO _____ (0)
YES _____ (1)
REFUSED _____ (98)
DON'T KNOW _____ (99)

PHYSICAL ACTIVITY

91. In the last three months, have you exercised for more than 30 minutes per day? Examples: walking, running, swimming, biking or playing soccer, etc…

NO _____ (0) (SKIP TO QUESTION 94)
YES _____ (1)
REFUSED _____ (98) (SKIP TO QUESTION 94)
DON'T KNOW _____ (99)

92. How long do you exercise for when you do it?

________ (Minutes) (Code Refused as 98, Don't Know as 99)

93. How frequently do you exercise?

________ (Times per week) (Code Refused as 98, Don't Know as 99)

94. How much time do you spend doing household chores at home?

________ (Minutes per day) (Code Refused as 98, Don't Know as 99)

95. How many times do you do household chores per week?

________ (Times per week) (Code Refused as 98, Don't Know as 99)

CURRENT PREGNANCY AND EXAM DATA

96. Fetal Heart Rate _____ beats per minute (bpm)

97. Pre-Gestational Weight ___ ___ ___. ___ lbs

98. Current weight of the mother: ___ ___ ___ . ___ lbs.

99. Current Height: _______ cm

100. Uterine height: _______ cm

101. Current Waist Circumference: _______ cm

102. Current Abdominal Circumference: _______ cm

103. Current Arm Circumference: _______ cm

104. Biceps Skinfold: _______ cm

105. Triceps Skinfold: _______ cm

106. Subscapular Skinfold: _______ cm

107. Blood Pressure:

107a. Systolic __________ mmHg

107b. Diastolic __________ mmHg

108. Temperature _______°C

109. Mark the research samples that were taken or will be taken today:

Samples were taken = 1   *Samples were not taken = 0*

a. Urine _______________
b. Blood _______________

110. Time at the end of the interview ___ ___   ___ ___
HRS MIN

Date and time of the next scheduled visit: _________________________

In the next visit we will be evaluating the use of cleaning products in your house. Also, we will like to see the medications and pesticides that you are using, if any. Please, have them handy in your house so that we can revise their contents in our next visit. Thanks.

Código del Lugar: ___<br><br>Sj: San Juan UMC<br>Ar: Arecibo<br>Ma: Manatí<br><br>ID: ________________<br><br>Visita #: V1	**PRoTECT**<br>(Puerto Rico como Lugar para Explorar Amenazas por Contaminación Ambiental)<br><br>**Primera Visita (~20 Semanas) Cuestionario**	Hora de Inicio:<br><br>Hora: __ __ Hr. __ __ Min.<br><br>FECHA:<br>__ __ / __ __ / __ __ __ __<br>MM DD YYYY<br><br>ENTREVISTADOR:<br>________________________

Introducción:

Buenos días (tardes). Gracias por acceder a reunirse con nosotros. Me gustaría preguntarle algunas preguntas sobre sus antecedentes: donde ha vivido, trabajos que ha tenido, su historial médico y sus hábitos personales y alimenticios. Algunas de estas preguntas pueden ser sensitivas en su naturaleza, así que no tiene que responder si así escoge. Estamos tratando de aprender cómo estas cosas pueden afectar la salud. Agradeceremos su ayuda al responder estas preguntas lo mejor que pueda. Pero, no tiene que contestar cualquier pregunta que usted no desee. Todo lo que me diga será mantenido en privado y no será divulgado con nadie fuera del estudio.

Durante la entrevista, por favor no dude en pedirme que sea un poco más lento o que repita cualquier pregunta que no esté clara. Antes de comenzar, quiero recordarle que todo lo que me diga será estrictamente confidencial.

---

1. ¿Cuál es su nombre completo?

______________________	______________________	______________________	______________________
NOMBRE (S)	SEGUNDO NOMBRE	PATERNO	MATERNO

2. Fecha de Nacimiento (mes/día/año) __ __ /__ __ /__ __

3. ¿Cuál es su fecha estimado de parto?

|___|___| / |___|___| / |___|___|
MES DÍA AÑO

REHÚSA.. 98
NO SABE.. 99

Edad Gestacional:

a. Fecha Última Menstruación (FUM) __ __/__ __/__ __
(mes /día / año)

b. Edad Gestacional por Ultra Sonido (USG) _____Semanas _____ Días

4. ¿Ha cambiado de dirección residencial desde nuestro último encuentro?

Sí 1
No .... 0 (CONTINÚE A LA PREGUNTA 6)

5. ¿Cuál es su dirección actual?

Calle___________________________________ No._________

Urb./Edif. _______________________________ Apt. # ______

Ciudad ____ Código Postal _________

Referencias (entrecalles, cruces o área de interés):

____

6. Lugar de origen (Ciudad y estado correspondiente)

A. ¿Dónde nació usted? ____________________________________

B. ¿Dónde nació su madre? _________________________________

C. ¿Dónde nació su padre? __________________________________

INFORMACIÓN DEMOGRÁFICA

7. Estado civil:

Soltera	_____ (1)
Casada	_____ (2)
Divorciada	_____ (3)
Viuda	_____ (4)
Unión Libre	_____ (5)
REHÚSA	_____ (98)
NO SABE	_____ (99)

8. ¿Se considera usted Hispana o Latina?

______ SÍ	1	
______ No	0	(CONTINÚE A LA PREGUNTA 10)

9. ¿Cuál de los siguientes representa su origen o ascendencia hispana?

______ PUERTORRIQUEÑA	1
______ CUBANA/CUBANA-AMERICANA	2
______ DOMINICANA	3
______ MEXICANA	4
______ MEXICANA-AMERICANA...	5
______ CENTRO/SURAMERICANA	6
______ OTRA (ESPECIFIQUE):__________________________________	97
______ REHÚSA	98
______ NO SABE	99

10. ¿Qué raza usted se considera? Puede seleccionar una o más. (No-0, Sí-1, Rehúsa-98, No sabe 99)

a. Blanca,.._____
b. Negra o Afro-Americana, ..._____
c. Indígena Americana o Indígena de Alaska, ..._____
d. Asiática ...._____
e. Nativa de Hawái u Otras Islas Pacíficas ...._____
f. Mestiza ...._____
g. ALGUNA OTRA RAZA (ESPECIFIQUE): _______________________

11. ¿Cuál es el grado o nivel de escolaridad más alto que ha completado?

______ NO ESCUELA.. 01

ELEMENTAL

______ PRE-ESCOLAR A 4to GRADO.. 02
______ 5to - 6to GRADO 03
______ 7mo -8vo GRADO 04

ESCUELA SUPERIOR

______ 9no GRADO 05
______ 10mo GRADO 06
______ 11 GRADO... 07
______ 12 GRADO (NO DIPLOMA) 08
______ DIPLOMA ESCUELA SUPERIOR 09
______ GED O EQUIVALENTE 10

UNIVERSIDAD

______ ALGUNOS CRÉDITOS UNIVERSITARIOS, PERO MENOS DE UN AÑO 11
______ 1 O MÁS AÑOS DE UNIVERSIDAD, PERO SIN GRADO.......... 12
______ GRADO ASOCIADO: OCUPACIONAL, TÉCNICO, O
______ PROGRAMA VOCACIONAL.. 13
______ GRADO ASOCIADO: PROGRAMA ACADÉMICO 14
______ GRADO BACHILLERATO (Ej.: BA, BS) 15

ESCUELA GRADUADA

______ MAESTRÍA (Ej.: MA, MS, MSW, MEng, MBA)........................... 16
______ GRADO DE ESCUELA PROFESIONAL (Ej. MD, DDS, DVM, JD)17
______ GRADO DOCTORAL (Ej., Ph.D., Ed.D)…………………………… 18
______ REHÚSA 98
______ NO SABE 99

Ahora voy a hacerle algunas preguntas sobre el patrimonio del bebé en el lado del padre.

12. ¿Considera usted que el padre de su bebé es hispano o latino?

Sí 1
No ....….. 0 (CONTINÚE A LA PREGUNTA 14)
REHÚSA 98
NO SABE 99

13. ¿Cuál de las siguientes opciones representa el origen o ascendencia hispana del padre?

______ PUERTORRIQUEÑO... 1
______ CUBANO/CUBANO-AMERICANO 2
______ DOMINICANO... 3
______ MEXICANO... 4
______ MEXICANO-AMERICANO 5
______ CENTRO/SURAMERICANO 6
______ OTRO (ESPECIFIQUE):__________________________________ 97
______ REHÚSA 98
______ NO SABE 99

14. ¿De qué raza usted considera al padre de su bebé? Usted puede seleccionar uno o más respuestas. (No-0, Sí-1, Rehúsa-98, No sabe 99)

a. Blanco,.._____
b. Negro o Afro-Americano, ..._____
c. Indígena Americano o Indígena de Alaska, ..._____
d. Asiático ...._____
e. Nativo de Hawái u Otras Islas Pacíficas ...._____
f. Mestizo……………………………………………………………………..._____
g. ALGUNA OTRA RAZA (ESPECIFIQUE): _____________________

15. ¿Cuál es el grado o nivel de escolaridad más alto que ha completado su esposo/pareja?

______ NO ESCUELA.. 01

ELEMENTAL
______ PRE-ESCOLAR A 4to GRADO.. 02
______ 5 ^to - 6 ^to GRADO 03
______ 7mo -8 ^vo GRADO 04

ESCUELA SUPERIOR
______ 9no GRADO 05
______ 10mo GRADO 06
______ 11 GRADO.. 07
______ 12 GRADO (NO DIPLOMA) 08
______ DIPLOMA ESCUELA SUPERIOR 09
______ GED O EQUIVALENTE 10

UNIVERSIDAD
______ ALGUNOS CRÉDITOS UNIVERSITARIOS, PERO MENOS DE UN AÑO..11
______ 1 Ó MÁS AÑOS DE UNIVERSIDAD, PERO SIN GRADO……... 12
______ GRADO ASOCIADO: OCUPACIONAL, TÉCNICO, O
______ PROGRAMA VOCACIONAL... 13
______ GRADO ASOCIADO: PROGRAMA ACADÉMICO 14
______ GRADO BACHILLERATO (Ej.:, BA, BS) 15

ESCUELA GRADUADA
______ MAESTRÍA (Ej.:, MA, MS, MSW, MEng, MBA)........................... 16
______ GRADO DE ESCUELA PROFESIONAL (Ej.:, MD, DDS, DVM, JD)17
______ GRADO DOCTORAL (Ej.:, Ph.D., Ed.D.) ………………………… 18
______ REHÚSA ……………………………………………………………… 98
______ NO SABE ……………………………………………………………….. 99

ESTADO DE EMPLEO

Ahora me gustaría hacerle unas preguntas acerca de cualquier trabajo escolar, empleo, trabajo voluntario, y pasatiempos que ha realizado recientemente. ***Por favor incluya aquellas actividades que usted hace o ha hecho por lo menos 4 horas por semana.***

16. ¿Es usted actualmente una estudiante a tiempo completo o parcial? Esto incluye escuela vocacional o técnica que no sea conducida en un salón de clases.

_____ NO, NO SOY ESTUDIANTE .... 0 (CONTINÚE A LA PREGUNTA 20)
_____ SÍ, ESTUDIANTE TIEMPO COMPLETO 1
_____ SÍ, ESTUDIANTE TIEMPO PARCIAL... 2
_____ REHÚSA... 98 (CONTINÚE A LA PREGUNTA 20)
_____ NO SABE... 99

17. ¿Qué tipo o tipos de escuela está actualmente atendiendo?

_____ ESCUELA SUPERIOR 1
_____ ESCUELA TÉCNICA 2
_____ COLEGIO O UNIVERSIDAD 3
_____ ESCUELA GRADUADA 4
_____ ESCUELA PROFESIONAL (Ej.: MEDICINA, LEYES, DENTAL) ... 5
_____ OTRAS (ESPECIFIQUE): ______________________________ 97
_____ REHÚSA 98
_____ NO SABE 99

18. ¿Qué mejor describe el lugar donde normalmente vas a la escuela? Seleccione todas las que apliquen. (No-0, Sí-1, Rehúsa-98, No sabe 99)

a. SALÓN DE CLASES ...._____
b. RESIDENCIA, COMO SU CASA O LA DE ALGUIEN ...._____
c. LABORATORIO ...._____
d. GARAJE O TALLER ...._____
e. VEHÍCULO DE MOTOR ...._____
f. ALGUNA OTRA LOCALIDAD (ESPECIFIQUE): ________________

19. (Por favor dígame la dirección donde actualmente atiende escuela más frecuente)

Calle____________________________________ No._________

Urb./Edif. ____________________________Apt. # ___________

Ciudad ______________________ Código Postal _________

Referencia: (cruce o punto de interés): ____

REHÚSA 98
NO SABE 99

20. Ahora nos gustaría preguntar acerca de sus trabajos que ha tenido recientemente.
Desde que quedó embarazada,

	NÚMERO	REH	NS
a. ¿Cuántos trabajos a tiempo complete ha tenido?	|___|___|	98	99
b. ¿Cuántos trabajos a tiempo parcial ha tenido?	|___|___|	98	99
c. ¿Cuántos trabajos voluntarios ha tenido (departamento bomberos, Sociedad Protectora de Animales, etc.)?	|___|___|	98	99

21. ¿Está usted actualmente empleada?

_____ SÍ 1
_____ NO .... 0 (CONTINÚE A LA PREGUNTA 35)
_____ REHÚSA .... 98 (CONTINÚE A LA PREGUNTA 35)
_____ NO SABE .... 99 (CONTINÚE A LA PREGUNTA 35)

22. ¿Para este trabajo, cuál es su título o la ocupación?

____
TÍTULO

_____ REHÚSA 98
_____ NO SABE 99

23. ¿Para este trabajo, quién es su empleador?

____
EMPLEADOR/PATRÓN

_____ REHÚSA.. 98
_____ NO SABE.. 99

24. ¿Qué tipo de actividades usted hace más frecuente en este trabajo? Por ejemplo, dar clases, trabajar en una computadora, mantener los libros de contabilidad, archivar, fotocopiar, contestar el teléfono, mesera, ayuda al cliente, trabajo de laboratorio, o carpintería. (EXPLORE: ¿Alguna otra cosa?)

____
ACTIVIDAD

_____ REHÚSA.. 98
_____ NO SABE.. 99

25. ¿En qué tipo de institución, negocios o industria es este trabajo? Eso es, qué manufactura o hace la compañía o institución.

____
INDUSTRIA

_____ REHÚSA.. 98
_____ NO SABE.. 99

26. ¿En promedio, cuántas horas a la semana usualmente trabaja en esta industria?

|___|___|___|
NÚMERO DE HORAS

_____ REHÚSA.. 98
_____ NO SABE.. 99

27. ¿Cuál es su turno de trabajo?

_____ Turno en el día.. 1
_____ Turno en la tarde... 2
_____ Turno en la noche.. 3
_____ Turno rotativos.. 4
_____ REHÚSA.. 98
_____ NO SABE.. 99

28. ¿Cuán frecuente usted levanta o mueve objetos pesados en el trabajo?

_____ Nunca 1
_____ Algunas veces 2
_____ Frecuentemente 3
_____ REHÚSA.. 98
_____ NO SABE.. 99

29. ¿Cuál es el nivel de exerción física en su trabajo?

_____ Leve 1
_____ Moderada 2
_____ Fuerte 3
_____ REHÚSA.. 98
_____ NO SABE.. 99

30. ¿Cuál es la temperatura regular de su lugar de trabajo?

_____ Normal (68-73°F) temperatura ambiente 1
_____ Sobre temperatura ambiente 2
_____ Por debajo de la temperatura ambiente....................................... 3
_____ REHÚSA.. 98
_____ NO SABE... 99

31. ¿Cuál de los siguientes describe el lugar donde típicamente labora en su trabajo? Seleccione todas las que apliquen. (No-0, Sí-1, Rehúsa-98, No sabe 99)

a. ÁREA DE OFICINA..._____
b. TIENDA.._____
c. SALÓN DE CLASE ...._____
d. HOTEL O MOTEL..._____
e. RESTAURANTE ...._____
f. RESIDENCIA, TALES COMO SU CASA O LA DE ALGUIEN MÁS ....._____
g. CENTRO DE SALUD U HOSPITAL.._____
h. LABORATORIO ...._____
i. FÁBRICA, PLANTA, O ÁREA DE PRODUCCION............................._____
j. ALMACÉN..._____
k. GARAJE O TALLER ...._____
l. SALÓN.._____
m. MUELLE DE CARGA.._____
n. CONSTRUCCIÓN.._____
o. JARDINES, PATIOS, O SUELOS.._____
p. GRANJA, CAMPO, O CORRALES.._____
q. VEHÍCULO DE MOTOR ...._____
r. ALGÚN OTRA LUGAR (ESPECIFIQUE):______________________

32. ¿Cuál es la dirección donde actualmente trabaja?

_____ CASA 1
_____ VARÍA (CONSTRUCCIÓN, PAISAJISMO) 2
_____ TENGO DIRECCIÓN EXACTA.. 3
_____ OTRAS (ESPECIFIQUE): _______________________________ 97
_____ REHÚSA.. 98
_____ NO SABE... 99

33. ¿Ha cambiado de dirección de su trabajo desde nuestro último encuentro?

_____ Sí 1
_____ No .... ….. 0 (CONTINÚE A LA PREGUNTA 35)

34. Por favor dígame la dirección donde actualmente trabaja.

Calle_____________________________________ No._________

Urb./Edif. ______________________________Apt. # ___________

Ciudad ______________________ Código Postal _________

Referencia: (cruce o punto de interés): ____

_____ REHÚSA.. 98
_____ NO SABE... 99

INGRESO

35. De estos grupos de ingresos, ¿qué categoría mejor representa sus ingresos por hogar o familia en el año pasado? Recuerde, una familia es un grupo de dos o más personas que vivan juntas y que estén relacionadas por nacimiento, matrimonio, o adopción.

_____ Menos de $4,999 01
_____ $5,000-$9,999.......... 02
_____ $10,000-$19,999.......... 03
_____ $20,000-$29,999.......... 04
_____ $30,000-$39,999.......... 05
_____ $40,000-$49,999.......... 06
_____ $50,000-$74,999.......... 07
_____ $75,000-$99,999.......... 08
_____ $100,000-$199,000.......... 09
_____ $200,000 o más 10
_____ REHÚSA.......... 98
_____ NO SABE.......... 99

36. ¿Hay algún miembro de la familia que no viva en su hogar que también esté siendo apoyado por este ingreso?

_____ Sí 1
_____ No .... 2 (CONTINÚE A LA PREGUNTA 38)
_____ REHÚSA.......... 98 (CONTINÚE A LA PREGUNTA 38)
_____ NO SABE.......... 99 (CONTINÚE A LA PREGUNTA 38)

37. ¿Cuántos otros miembros de la familia, que no vivan en su hogar, están apoyados por este ingreso?

|___|___|
NÚMERO
_____ REHÚSA.......... 98
_____ NO SABE.......... 99

TABAQUISMO

Ahora me gustaría hacerle algunas preguntas sobre el uso del tabaco.

38. ¿Alguna vez en su vida ha fumado?

_____ Sí 1
_____ No .... 0 (CONTINÚE A LA PREGUNTA 47)
_____ REHÚSA 98
_____ NO SABE 99

39. ¿Actualmente fuma?

_____ Sí 1
_____ No 0
_____ REHÚSA 98
_____ NO SABE 99

40. ¿En su vida, ha fumado más de 100 cigarrillos?

_____ Sí 1
_____ No 0
_____ REHÚSA 98
_____ NO SABE 99

41. ¿Hubo alguna vez un momento en donde se fumaba al menos un cigarrillo al día por un mes o más de un mes?

_____ Sí 1
_____ No 0
_____ REHÚSA 98
_____ NO SABE 99

42. ¿Por cuántos años diría que se fumó al menos un cigarrillo diario?

___ ___ Años (Código: REHÚSA-98, NO SABE-99)

43. ¿Durante el tiempo que fumaba al menos un cigarrillo al día, cuántos cigarrillos al día fumaba en promedio?

___ ___ cigarrillos/ día (Código: REHÚSA-98, NO SABE-99)

44. ¿Cuándo fue su último cigarrillo?

Hoy	______	(0)
Semana pasada	______	(1)
Más de una semana	______	(2)
Más de un mes	______	(3)
Antes del Embarazo	______	(4)
Rehúsa	______	(98)
No Sabe	______	(99)

45. ¿Ha dejado de fumar por 6 meses o más?

_____ Sí 1
_____ No 0
_____ REHÚSA 98
_____ NO SABE 99

45a. ¿Dejó de fumar debido a su embarazo?

_____ Sí 1
_____ No 0
_____ REHÚSA 98
_____ NO SABE 99

46. ¿Si dejó de fumar y comenzó de nuevo, por cuantos años no fumó?

____ ____ Años (Código: REHÚSA-98, NO SABE-99)

47. ¿Alguien en su hogar fuma a diario?

_____ Sí 1
_____ No .... 0 (CONTINÚE A PREGUNTA 47)
_____ REHÚSA 98
_____ NO SABE 99

47a. ¿Quién fuma en su hogar? ¿Cuántos cigarrillos fuman en promedio? Seleccione todas las que apliquen. (No-0, Sí-1, Rehúsa-98, No sabe 99)

a. Padre (Abuelo del bebé)	____	____ cigarrillos/ día promedio
b. Madre (Abuela del bebé)	____	____ cigarrillos/ día promedio
c. Cónyuge	____	____ cigarrillos/ día promedio
d. Hermano(a)	____	____ cigarrillos/ día promedio
e. Otro	____	____ cigarrillos/ día promedio

48. Desde que quedó embarazada, en promedio, cuántas horas al día ha estado alrededor de alguien que fuma, lo suficientemente cerca para que pueda oler el humo?

____ ____ horas/día (Código: REHÚSA-98, NO SABE-99)

USO DE ALCOHOL

Las próximas tres preguntas son acerca del consumo de alcohol. Cuando pregunto por la cantidad de tragos, me refiero a una botella de 12 onzas de cerveza, a una copa de vino de 4 onzas, y un trago de licor son considerados como un trago. También, las preguntas cuestionan los patrones típicos de consumo de alcohol, en vez del consumo ocasional (en fiestas o celebraciones, por ejemplo).

49. ¿Cuándo fue la última vez que tomó una bebida alcohólica?

No bebo alcohol	______	(0)	(CONTINÚE A LA PREGUNTA 52)
Hoy	______	(1)	
Ayer	______	(2)	
Semana pasada	______	(3)	
Más de una semana	______	(4)	
Más de un mes	______	(5)	
Antes del Embarazo	______	(6)	
Rehúsa	______	(98)	
No Sabe	______	(99)	

50. Durante el pasado año, ¿cuántos **días a la semana** consumía bebidas alcohólicas?

Ninguno	______	(0)
1 a 2 días	______	(1)
3 ó más días	______	(2)
Rehúsa	______	(98)
No Sabe	______	(99)

51. Durante el año pasado, cada vez que consumía alcohol, ¿cuántos tragos usualmente ingería?

Ninguno	______	(0)
1 a 2 tragos	______	(1)
3 ó 5 tragos	______	(2)
6 o más tragos	______	(3)
Rehúsa	______	(98)
No Sabe	______	(99)

USO DE DROGAS

A continuación, voy a hacerle unas preguntas acerca de las drogas que pueda tomar. Quiero recordarle que todo lo que usted diga será totalmente confidencial y no será compartido con nadie.

52. ¿Está usted actualmente fumando **marihuana**?

_____ Sí 1
_____ No 0
_____ REHÚSA 98
_____ NO SABE 99

53. ¿Está usted actualmente utilizando otra droga ilícita, incluyendo drogas que se inyectan o se fuman?

_____ Sí 1
_____ No .... 0 (CONTINÚE A LA PREGUNTA 53)
_____ REHÚSA .... 98 (CONTINÚE A LA PREGUNTA 53)
_____ NO SABE .... 99 (CONTINÚE A LA PREGUNTA 53)

53a. ¿Cuáles son dichas drogas? (Código: REHÚSA-98, NO SABE-99)

I. ____________________
II. ____________________
III. ____________________
IV. ____________________
V. ____________________

USO DE AGUA

A continuación, quisiera preguntarle acerca del uso de agua.

54. ¿Cuál es la fuente principal de agua en su casa que utiliza para beber?

Autoridad de Acueductos y Alcantarillados de PR (AAA)	______ (1)
Pozo privado	______ (2)
Pozo Comunitario (No-AAA)	______ (3)
Toma agua superficial – No-AAA	______ (4)
Cisterna (Tanque de Agua)	______ (5)
Colección de Lluvia	______ (6)
Agua embotellada	______ (7)
Rehúsa	______ (98)
No Sabe	______ (99)

55. ¿Cuál es la fuente principal de agua en su casa que utiliza para cocinar?

Autoridad de Acueductos y Alcantarillados de PR (AAA)	______ (1)
Pozo privado	______ (2)
Pozo Comunitario (No-AAA)	______ (3)
Toma agua superficial – No-AAA	______ (4)
Cisterna (Tanque de Agua)	______ (5)
Colección de Lluvia	______ (6)
Agua embotellada	______ (7)
Rehúsa	______ (98)
No Sabe	______ (99)

56. ¿Usted almacena el agua que utiliza para beber y cocinar en su residencia (en especial, usa una cisterna)?

_____ Sí 1
_____ No .... 0 (CONTINÚE A LA PREGUNTA 56)
_____ REHÚSA .... 98 (CONTINÚE A LA PREGUNTA 56)
_____ NO SABE .... 99 (CONTINÚE A LA PREGUNTA 56)

56a. ¿De qué está hecho el contenedor?

Plástico	____ (1)
Metal	____ (2)
Otro	____ (3)
Rehúsa	____ (98)
No Sabe	____ (99)

57. ¿Utiliza algún tipo de tratamiento en el agua para beber en su hogar?

_____ Sí 1
_____ No .... 0 (CONTINÚE A LA PREGUNTA 57)
_____ REHÚSA .... 98 (CONTINÚE A LA PREGUNTA 57)
_____ NO SABE .... 99 (CONTINÚE A LA PREGUNTA 57)

57a. Si trata el agua, ¿Qué tipo de tratamiento?

Filtro de Cartucho _____ (1)
Osmosis Reversible _____ (2)
Hierve el agua _____ (3)
Suavizador de Agua _____ (4)
Rehúsa _____ (98)
No Sabe _____ (99)

58. ¿Cuán frecuente usted bebe agua filtrada o tratada?

Casi nunca o Nunca _____ (1)
Aproximadamente ¼ del tiempo _____ (2)
Aproximadamente ½ del tiempo _____ (3)
Alrededor de ¾ del tiempo _____ (4)
Casi siempre o Siempre _____ (5)
Rehúsa _____ (98)
No Sabe _____ (99)

HISTORIAL MÉDICO PERSONAL

Ahora le estaré hacienda unas preguntas relacionadas a su estado actual de salud.

¿Usted presenta alguno de los siguientes síntomas?

Favor de Indicar (No-0, Sí-1, Rehúsa- 98, No Sabe - 99)

59. Dolor de cabeza _____ Sí _____ No 58a. Episodios por día: [1-2] ___(1), [2-4] ___(2), [> 4] ___(3)

60. Mareo _____ Sí _____ No 59a. Episodios por día: [1-2] ___(1), [2-4] ___(2), [> 4] ___(3)

61. Náusea _____ Sí _____ No 60a. Episodios por día: [1-2] ___(1), [2-4] ___(2), [> 4] ___(3)

62. Vómito diario _____ Sí _____ No 61a. Episodios por día: [1-2] ___(1), [2-4] ___(2), [> 4] ___(3)

63. Escucha zumbido _____ Sí _____ No 62a. Episodios por día: [1-2] ___(1), [2-4] ___(2), [> 4] ___(3)

64. Ve lucecitas _____ Sí _____ No 63a. Episodios por día: [1-2] ___(1), [2-4] ___(2), [> 4] ___(3)

65. Movimiento fetal _____ Sí _____ No

65a. El movimiento es como de costumbre (es decir el tiempo del día o frecuencia)? (1) Sí ____ (0) No ____

65b. Si no a la (65a) ¿Cree usted que los movimientos han disminuido? (1) Sí ____ (0) No ____

66. Contracciones _____ Sí _____ No (SI CONTESTA NO, CONTINÚE A LA PREGUNTA 68)

67. Si las presenta, ¿qué tan frecuentes son?

_______ en una semana , __________ en un día _________ en una hora, ___________ en 10 minutos

68. Salida de líquido/sangre/flujo por la vagina _____ Sí _____ No (SI CONTESTA NO, CONTINÚE A LA PREGUNTA 69)

68a. ¿Qué características tiene el flujo vaginal? (Marque todas las que apliquen)

I. Transparente _____ Sí _____ No

II. Blanco _____ Sí _____ No

III. Grumoso _____ Sí _____ No

IV. Maloliente _____ Sí _____ No

V. Amarillo _____ Sí _____ No

VI. Verde _____ Sí _____ No

VII. Café _____ Sí _____ No

VIII. Sangre roja brillante _____ Sí _____ No

69. Ardor al orinar _____ Sí _____ No

70. Mayor frecuencia que la acostumbrada para orinar _____ Sí _____ No

71. Pies hinchados _____ Sí _____ No

72. Actualmente, diría usted que su estado de salud general es:

_____ Excelente, ... 1
_____ Muy Buena, ... 2
_____ Buena, ... 3
_____ Razonable 4
_____ Pobre 5
_____ REHÚSA 98
_____ NO SABE 99

73. ¿Cuántos empastes dentales tiene usted?

|___|___|
#Empastes

_____ REHÚSA .... 98 (CONTINÚE A LA PREGUNTA 74)
_____ NO SABE .... 99 (CONTINÚE A LA PREGUNTA 74)

74. Cuántos de estos empastes son de color plata?

|___|___|
#Empastes

_____ REHÚSA 98
_____ NO SABE 99

HISTORIAL DE EMBARAZOS

75. Con respecto a este embarazo, ¿estaba intentando quedar embarazada?

_____ Sí 1
_____ No .... 0 (CONTINÚE A LA PREGUNTA 77)
_____ REHÚSA 98
_____ NO SABE 99

76. ¿Por cuántos meses, estuvo intentando quedar embarazada? (Nota a entrevistador: Si la respuesta es 0 menos, anote 0 y continúe a la pregunta 78)

|___|___|
MESES

_____ REHÚSA 98
_____ NO SABE 99

77. ¿Le habían dado alguna de las siguientes drogas para mejorar su ovulación para **este embarazo**? Seleccione todas las que apliquen. (No-0, Sí-1, Rehúsa-98, No sabe 99)

a. CLOMID ...._____
b. GONAL F ...._____
c. BRAVELLE ...._____
d. FOLLISTIM ...._____
e. REPRONEX ...._____
f. PERGONAL ...._____
g. PREGNYL ...._____
h. PROFASI ...._____
i. NOVAREL ...._____
j. OTRAS DROGAS (ESPECIFIQUE):___________________________

78. ¿Cuál era su peso antes de quedar embarazada?

|___|___|___|
LIBRAS (1)

_____ REHÚSA.. 98
_____ NO SABE.. 99

79. ¿Qué edad tenía cuando tuvo su primera menstruación?

|___|___|
EDAD

_____ REHÚSA.. 98
_____ NO SABE.. 99

80. ¿Cuántos embarazos ha tenido? (Incluyendo éste)

|___|___| Embarazos (NOTA A ENTREVISTADOR: SI LA PARTICIPANTE CONTESTA 1, CONTINÚE A LA PREGUNTA 88).

_____ REHÚSA.. 98
_____ NO SABE.. 99

81. ¿Cuántos nacimientos vivos ha tenido? (no incluyendo éste)

|___|___| Nacimientos Vivos

_____ REHÚSA……………... 98
_____ NO SABE.. 99

82. ¿Qué edad tenía cuando dio a luz por primera vez?

|___|___| Años (Código – Rehúsa-98 o No Sabe-99)

83. ¿Han sido alguno de sus embarazos previos por cesárea?

_____ Sí 1
_____ No 0
_____ REHÚSA.. 98
_____ NO SABE.. 99

83a. ¿Cuántas cesáreas han sido realizadas?

|___|___| cesáreas (Código – Rehúsa-98 o No Sabe-99)

83b. ¿Cuántas veces ha tenido un aborto espontáneo?

|___|___| veces (Código – Rehúsa-98 o No Sabe-99)

84. ¿Cuántos niños ha tenido que hayan pesado menos de 5.5 lbs. al momento de nacer o que el doctor le haya mencionado que es de bajo peso?

|___|___| Niños (Código – Rehúsa-98 o No Sabe-99)

85. ¿Cuántos niños ha tenido que hayan nacido antes de la semana 37 o que el doctor le haya mencionado que es prematuro?

|___|___| Niños (Código – Rehúsa-98 o No Sabe-99)

86. ¿Cuántos de sus embarazos han sido natimuertos?

|___|___| Número (Código – Rehúsa-98 o No Sabe-99)

87. Durante sus embarazos previos, ¿tuvo usted alguna de las siguientes complicaciones?
Favor de Indicar (No-0, Sí-1, Rehúsa- 98, No Sabe - 99)

a. No Aplica (Primer embarazo) ______ (CONTINÚE A LA PREGUNTA 87)
b. Sangrando 1er Trimestre ______
c. Sangrado 2do Trimestre ______
d. Sangrado 3er Trimestre ______
e. Ruptura prematura de las membranas (RPM) ______
f. Pre-eclampsia ______
g. Eclampsia ______
h. Cardiopatía ______
i. Neuropatía ______
j. Diabetes Gestacional ______
k. Anemia ______
l. Infecciones Vaginales ______
m. Enfermedades de Transmisión Sexual ______
n. Ninguna Complicación ______
m. Otras (Especifique) ______________________________

88. ¿Ha tenido alguna compilación en este embarazo? Favor de Indicar (No-0, Sí-1, Rehúsa- 98, No Sabe - 99)

a. Sangrado ______
b. Anemia ______
c. Infecciones Vaginales ______
d. Infecciones del tracto urinario ______
e. Enfermedades de Transmisión Sexual ______
f. Ruptura prematura de las membranas (RPM) ______
g. Pre-eclampsia ______
h. Eclampsia ______
i. Cardiopatía ______
j. Neuropatía ______
k. Diabetes Gestacional ______

89. ¿Cuántas semanas de embarazo tenía cuando se enteró que estaba embarazada?

_______ (Semanas) (Código – Rehúsa-98 o No Sabe-99)

90. ¿Cuándo comenzó el cuidado prenatal bajo la supervisión de un profesional de la salud?

___ ___/ ___ ___/___ ___ (Código – Rehúsa-98 o No Sabe-99)
Mes / Día / Año

SUPLEMENTOS DIETÉTICOS

91. ¿En los 3 meses antes de que quedara embarazada, tomó alguna prenatal o multivitamínico? Un multivitamínico es una píldora que contiene diferentes vitaminas y minerales, tales como One-A-Day o Centrum?

Sí 1
No 0
REHÚSA 98
NO SABE 99

ACTIVIDAD FÍSICA

92. En los últimos tres meses, ¿Se ha ejercitado más de 30 minutos al día?
    Ejemplos: caminar, correr, nadar, jugar fútbol, correr bicicleta, etc.…

_____ Sí .... 1 (CONTINÚE A LA PREGUNTA 95)
_____ No 0
_____ REHÚSA 98
_____ NO SABE 99

93. ¿Por cuánto tiempo se ejercita cada vez que lo hace?

________ (Minutos)

_____ REHÚSA 98
_____ NO SABE 99

94. ¿Con cuánta frecuencia usted realiza ejercicio?

_________ (Veces por semana)

_____ REHÚSA 98
_____ NO SABE 99

95. ¿Cuánto tiempo le dedica usted a sus labores domésticas?

__________ (Minutos al día)

_____ REHÚSA 98
_____ NO SABE 99

96. ¿Cuántas veces a la semana realiza labores domésticas? ___________ (Veces por semana)

_____ REHÚSA 98
_____ NO SABE 99

EMBARAZO ACTUAL Y RESULTADOS DEL EXAMEN FÍSICO

97. Ritmo FCF _____ latidos por minuto (lpm)

98. Peso Pre-Gestacional ___ ___ ___. ___ lbs

99. Peso actual de la madre: ___ ___ ___ . ___ lbs.

100. Estatura: _______ cm

101. Fondo Uterino ______ cm

102. Circunferencia actual (cintura): _______ cm

103. Circunferencia actual (abdominal): _______ cm

104. Circunferencia actual (media de brazo): _______ cm

105. Pliegue cutáneo bicipital: _______ cm

106. Pliegue cutáneo tricipital: _______ cm

107. Pliegue cutáneo subescapular: _______ cm

108. Presión Arterial:

108a. Sistólica ______ mmHg

108b. Diastólica ______ mmHg

109. Temperatura _______°C

110. Anote cuales muestras se tomaron o se tomarán el día de hoy:

Si tomaron la muestra = *1* No tomaron la muestra = *0*

A. Orina _______________
B. Sangre _______________

111. Hora al final de la entrevista ___ ___ ___ ___

HRS MIN

Fecha y hora de la próxima visita: ____

En la próxima visita estaremos evaluando los productos de limpieza que usted utiliza en su hogar. Además nos gustaría ver los medicamentos y pesticidas que esté usando si alguno. Por favor, téngalos a la mano en su casa para que revisemos su contenido en la próxima visita. Gracias.
