# Supplementary material for: Demographic risk factors for adverse birth outcomes in Puerto Rico in the PROTECT cohort
Source: PLoS One. 2019 Jun 13;14(6):e0217770. doi: 10.1371/journal.pone.0217770 (PMC6564423; doi:10.1371/journal.pone.0217770)
Supplement: S2 File — Table A. American College of Obstetricians and Gynecologists (ACOG) guidelines for redating gestational age based on ultrasonography. Adapted from ACOG Committee opinion Number 700, May 2017. Table B. Odds ratios (OR) and 95% confidence intervals (CI) for preterm birth in association with demographic and pregnancy characteristics in the PROTECT cohort 2011–2017. Table C. Odds ratios (OR) and 95% confidence intervals (CI) for postterm birth in association with demographic and pregnancy characteristics in the PROTECT cohort 2011–2017. Table D. Adjusted1 odds ratios (OR) and 95% confidence intervals (CI) for preterm birth in association with demographic and pregnancy characteristics in the PROTECT cohort 2011–2017. Table E. Adjusted odds ratios (aOR) and 95% confidence intervals (CI) for postterm birth in association with demographic and pregnancy characteristics in the PROTECT cohort 2011–2017. Table F. Odds ratios (OR) and 95% confidence intervals (CI) for small for gestational age (SGA) birth compared to appropriate for gestational age birth (AGA) in association with demographic and pregnancy characteristics in the PROTECT cohort 2011–2017. Table G. Odds ratios (OR) and 95% confidence intervals (CI) for large for gestational age (LGA) birth compared to appropriate for gestational age birth (AGA) in association with demographic and pregnancy characteristics in the PROTECT cohort 2011–2017. Table H. Adjusted1 odds ratios (OR) and 95% confidence intervals (CI) for small for gestational age (SGA) birth compared to appropriate for gestational age birth (AGA) in association with demographic and pregnancy characteristics in the PROTECT cohort 2011–2017. Table I. Adjusted1 odds ratios (OR) and 95% confidence intervals (CI) for large for gestational age (LGA) birth compared to appropriate for gestational age birth (AGA) in association with demographic and pregnancy characteristics in the PROTECT cohort 2011–2017. (DOCX) [file pone.0217770.s002.docx]

| **S2 File Table A. American College of Obstetricians and Gynecologists (ACOG) guidelines for redating gestational age based on ultrasonography. Adapted from ACOG Committee opinion Number 700, May 2017.^1^** | | |
| --- | --- | --- |
| **Gestational age range** | **Discrepancy between US estimate and LMP estimate that supports redating** | **Number of PROTECT participants with dating US from this window** |
| <9 weeks | >5 days | 428 |
| 9 to <14 weeks | >7 days | 265 |
| 14 to <16 weeks | >7 days | 20 |
| 16 to <22 weeks | >10 days | 49 |
| 22 to <28 weeks | >14 days | 20 |

^1^American College of Obstetricians and Gynecologists. Committee opinion no 700: method for estimating due date. Obstetrics and Gynecology. 2017; 129:e150-4. Abbreviations: US, ultrasound.

| **S2 File Table B. Odds ratios (OR) and 95% confidence intervals (CI) for preterm birth in association with demographic and pregnancy characteristics in the PROTECT cohort 2011-2017.** | | | | |
| --- | --- | --- | --- | --- |
| **Characteristic** |  | Term: N (%) | Preterm: N (%) | OR (95% CI) |
| Age at enrollment | 18-24 | 331 (38.6) | 41 (38.3) | ref |
|  | 25-29 | 262 (30.5) | 30 (28.0) | 0.92 (0.56-1.52) |
|  | 30-34 | 168 (19.6) | 25 (23.4) | 1.20 (0.71-2.04) |
|  | ≥35 | 96 (11.2) | 11 (10.3) | 0.93 (0.46-1.87) |
| Pre-pregnancy BMI | <25 kg/m^2^ | 467 (54.4) | 47 (43.9) | ref |
|  | 25-30 kg/m^2^ | 225 (26.2) | 27 (25.2) | 1.19 (0.72-1.96) |
|  | >30 kg/m^2^ | 132 (15.4) | 23 (21.5) | 1.73 (1.01-2.96) |
| Household income | <$20,000 | 328 (38.2) | 57 (53.3) | ref |
|  | $20,000-$40,000 | 225 (26.2) | 20 (18.7) | 0.51 (0.30-0.88) |
|  | >$40,000 | 177 (20.6) | 19 (17.8) | 0.62 (0.36-1.07) |
| Education level | <High school | 59 (6.9) | 13 (12.1) | ref |
|  | High school/equivalent | 109 (12.7) | 18 (16.8) | 0.75 (0.34-1.64) |
|  | Some college/technical | 308 (35.9) | 40 (37.4) | 0.59 (0.30-1.17) |
|  | College degree or higher | 372 (43.4) | 35 (32.7) | 0.43 (0.21-0.85) |
| Employment status | Unemployed | 314 (36.6) | 50 (46.7) | ref |
|  | Employed | 531 (61.9) | 55 (51.4) | 0.65 (0.43-0.98) |
| Marital status | Single | 177 (20.6) | 20 (18.7) | ref |
|  | Married/cohabitating | 673 (78.4) | 86 (80.4) | 1.13 (0.68-1.89) |
| Smoking in pregnancy | No | 833 (97.1) | 106 (99.1) | ref |
|  | Yes | 16 (1.9) | 0 | Not estimated |
| Alcohol use in pregnancy | No | 795 (92.7) | 96 (89.7) | ref |
|  | Yes | 51 (5.9) | 8 (7.5) | 1.30 (0.60-2.82) |
| Gravidity | 0-1 previous pregnancy | 337 (39.3) | 41 (38.3) | ref |
|  | >1 previous pregnancy | 512 (59.7) | 65 (60.7) | 1.04 (0.69-1.58) |
| Parity | No previous births | 407 (47.4) | 44 (41.1) | ref |
|  | ≥1 previous birth | 442 (51.5) | 62 (57.9) | 1.30 (0.86-1.95) |
| Mode of delivery | Vaginal | 459 (53.5) | 40 (37.4) | ref |
|  | C-section | 382 (44.5) | 58 (54.2) | 1.74 (1.14-2.67) |
| Infant sex | Male | 434 (50.6) | 57 (53.3) | ref |
|  | Female | 406 (47.3) | 41 (38.3) | 0.77 (0.50-1.17) |
| Season of delivery | November-June | 577 (67.2) | 70 (65.4) | ref |
|  | July-October | 281 (32.8) | 37 (34.6) | 1.09 (0.71-1.66) |
| Year of delivery | 2011 | 21 (2.4) | 4 (3.7) | 1.58 (0.47-5.26) |
|  | 2012 | 138 (16.1) | 21 (19.6) | 1.26 (0.61-2.59) |
|  | 2013 | 177 (20.6) | 16 (15.0) | 0.75 (0.35-1.59) |
|  | 2014 | 116 (13.5) | 14 (13.1) | ref |
|  | 2015 | 154 (17.9) | 19 (17.8) | 1.02 (0.49-2.12) |
|  | 2016 | 183 (21.3) | 26 (24.3) | 1.18 (0.59-2.35) |
|  | 2017 | 69 (8.0) | 7 (6.5) | 0.84 (0.32-2.18) |

| **S2 File Table C. Odds ratios (OR) and 95% confidence intervals (CI) for postterm birth in association with demographic and pregnancy characteristics in the PROTECT cohort 2011-2017.** | | | | |
| --- | --- | --- | --- | --- |
| **Characteristic** |  | Term: N (%) | Postterm: N (%) | OR (95% CI) |
| Age at enrollment | 18-24 | 331 (38.6) | 21 (33.3) | ref |
|  | 25-29 | 262 (30.5) | 24 (38.1) | 1.44 (0.79-2.65) |
|  | 30-34 | 168 (19.6) | 16 (25.4) | 1.50 (0.76-2.95) |
|  | ≥35 | 96 (11.2) | 2 (3.2) | 0.33 (0.08-1.43) |
| Pre-pregnancy BMI | <25 kg/m^2^ | 467 (54.4) | 36 (57.1) | ref |
|  | 25-30 kg/m^2^ | 225 (26.2) | 14 (22.2) | 0.81 (0.43-1.53) |
|  | >30 kg/m^2^ | 132 (15.4) | 11 (17.5) | 1.08 (0.54-2.18) |
| Household income | <$20,000 | 328 (38.2) | 26 (41.3) | ref |
|  | $20,000-$40,000 | 225 (26.2) | 19 (30.2) | 1.07 (0.58-1.97) |
|  | >$40,000 | 177 (20.6) | 13 (20.6) | 0.93 (0.46-1.85) |
| Education level | <High school | 59 (6.9) | 5 (7.9) | ref |
|  | High school/equivalent | 109 (12.7) | 5 (7.9) | 0.54 (0.15-1.95) |
|  | Some college/technical | 308 (35.9) | 18 (28.6) | 0.69 (0.25-1.93) |
|  | College degree or higher | 372 (43.4) | 34 (54.0) | 1.08 (0.41-2.87) |
| Employment status | Unemployed | 314 (36.6) | 20 (31.7) | ref |
|  | Employed | 531 (61.9) | 43 (68.3) | 1.27 (0.73-2.20) |
| Marital status | Single | 177 (20.6) | 12 (19.0) | ref |
|  | Married/cohabitating | 673 (78.4) | 51 (81.0) | 1.12 (0.58-2.14) |
| Smoking in pregnancy | No | 833 (97.1) | 61 (96.8) | ref |
|  | Yes | 16 (1.9) | 2 (3.2) | 1.71 (0.38-7.60) |
| Alcohol use in pregnancy | No | 795 (92.7) | 58 (92.1) | ref |
|  | Yes | 51 (5.9) | 5 (7.9) | 1.34 (0.52-3.50) |
| Gravidity | 0-1 previous pregnancy | 337 (39.3) | 37 (58.7) | ref |
|  | >1 previous pregnancy | 512 (59.7) | 26 (41.3) | 0.46 (0.27-0.78) |
| Parity | No previous births | 407 (47.4) | 41 (65.1) | ref |
|  | ≥1 previous birth | 442 (51.5) | 22 (34.9) | 0.49 (0.29-0.84) |
| Mode of delivery | Vaginal | 459 (53.5) | 32 (50.8) | ref |
|  | C-section | 382 (44.5) | 30 (47.6) | 1.13 (0.67-1.89) |
| Infant sex | Male | 434 (50.6) | 36 (57.1) | ref |
|  | Female | 406 (47.3) | 26 (41.3) | 0.77 (0.46-1.30) |
| Season of delivery | November-June | 577 (67.2) | 41 (65.1) | ref |
|  | July-October | 281 (32.8) | 22 (34.9) | 1.10 (0.64-1.89) |
| Year of delivery | 2011 | 21 (2.4) | 1 (1.6) | 0.46 (0.06-3.73) |
|  | 2012 | 138 (16.1) | 11 (17.5) | 0.77 (0.33-1.81) |
|  | 2013 | 177 (20.6) | 8 (12.7) | 0.44 (0.17-1.10) |
|  | 2014 | 116 (13.5) | 12 (19.0) | ref |
|  | 2015 | 154 (17.9) | 7 (11.1) | 0.44 (0.17-1.15) |
|  | 2016 | 183 (21.3) | 17 (27.0) | 0.90 (0.41-1.95) |
|  | 2017 | 69 (8.0) | 7 (11.1) | 0.98 (0.37-2.61) |

| **S2 File Table D. Adjusted^1^ odds ratios (OR) and 95% confidence intervals (CI) for preterm birth in association with demographic and pregnancy characteristics in the PROTECT cohort 2011-2017.** | | | | |
| --- | --- | --- | --- | --- |
| **Characteristic** |  | Term: N (%) | Preterm: N (%) | OR (95% CI) |
| Age at enrollment | 18-24 | 331 (38.6) | 41 (38.3) | ref |
|  | 25-29 | 262 (30.5) | 30 (28.0) | 1.07 (0.61-1.88) |
|  | 30-34 | 168 (19.6) | 25 (23.4) | 1.54 (0.81-2.89) |
|  | ≥35 | 96 (11.2) | 11 (10.3) | 1.17 (0.52-2.61) |
| Pre-pregnancy BMI | <25 kg/m^2^ | 467 (54.4) | 47 (43.9) | ref |
|  | 25-30 kg/m^2^ | 225 (26.2) | 27 (25.2) | 1.15 (0.68-1.94) |
|  | >30 kg/m^2^ | 132 (15.4) | 23 (21.5) | 1.65 (0.94-2.88) |
| Household income | <$20,000 | 328 (38.2) | 57 (53.3) | ref |
|  | $20,000-$40,000 | 225 (26.2) | 20 (18.7) | 0.54 (0.29-1.01) |
|  | >$40,000 | 177 (20.6) | 19 (17.8) | 0.67 (0.34-1.35) |
| Education level | <High school | 59 (6.9) | 13 (12.1) | ref |
|  | High school/equivalent | 109 (12.7) | 18 (16.8) | 0.76 (0.33-1.74) |
|  | Some college/technical | 308 (35.9) | 40 (37.4) | 0.52 (0.25-1.10) |
|  | College degree or higher | 372 (43.4) | 35 (32.7) | 0.36 (0.16-0.82) |
| Employment status | Unemployed | 314 (36.6) | 50 (46.7) | ref |
|  | Employed | 531 (61.9) | 55 (51.4) | 0.80 (0.48-1.34) |
| Marital status | Single | 177 (20.6) | 20 (18.7) | ref |
|  | Married/cohabitating | 673 (78.4) | 86 (80.4) | 1.29 (0.73-2.27) |
| Smoking in pregnancy | No | 833 (97.1) | 106 (99.1) | ref |
|  | Yes | 16 (1.9) | 0 | Not estimated |
| Alcohol use in pregnancy | No | 795 (92.7) | 96 (89.7) | ref |
|  | Yes | 51 (5.9) | 8 (7.5) | 1.48 (0.66-3.34) |
| Gravidity | 0-1 previous pregnancy | 337 (39.3) | 41 (38.3) | ref |
|  | >1 previous pregnancy | 512 (59.7) | 65 (60.7) | 0.28 (0.08-1.03) |
| Parity | No previous births | 407 (47.4) | 44 (41.1) | ref |
|  | ≥1 previous birth | 442 (51.5) | 62 (57.9) | 3.44 (0.95-12.49) |
| Mode of delivery | Vaginal | 459 (53.5) | 40 (37.4) | ref |
|  | C-section | 382 (44.5) | 58 (54.2) | 1.72 (1.10-2.68) |
| Infant sex | Male | 434 (50.6) | 57 (53.3) | ref |
|  | Female | 406 (47.3) | 41 (38.3) | 0.81 (0.52-1.25) |
| Season of delivery | November-June | 577 (67.2) | 70 (65.4) | ref |
|  | July-October | 281 (32.8) | 37 (34.6) | 1.05 (0.67-1.63) |
| Year of delivery | 2011 | 21 (2.4) | 4 (3.7) | 1.48 (0.40-5.46) |
|  | 2012 | 138 (16.1) | 21 (19.6) | 1.23 (0.58-2.60) |
|  | 2013 | 177 (20.6) | 16 (15.0) | 0.70 (0.32-1.54) |
|  | 2014 | 116 (13.5) | 14 (13.1) | ref |
|  | 2015 | 154 (17.9) | 19 (17.8) | 1.01 (0.48-2.16) |
|  | 2016 | 183 (21.3) | 26 (24.3) | 0.95 (0.45-1.98) |
|  | 2017 | 69 (8.0) | 7 (6.5) | 0.44 (0.14-1.36) |

^1^Models mutually adjusted for all covariates.

| **S2 File Table E. Adjusted odds ratios (aOR) and 95% confidence intervals (CI) for postterm birth in association with demographic and pregnancy characteristics in the PROTECT cohort 2011-2017.** | | | | |
| --- | --- | --- | --- | --- |
| **Characteristic** |  | Term: N (%) | Postterm: N (%) | aOR (95% CI) |
| Age at enrollment | 18-24 | 331 (38.6) | 21 (33.3) | ref |
|  | 25-29 | 262 (30.5) | 24 (38.1) | 1.35 (0.66-2.75) |
|  | 30-34 | 168 (19.6) | 16 (25.4) | 1.60 (0.72-3.54) |
|  | ≥35 | 96 (11.2) | 2 (3.2) | 0.36 (0.08-1.67) |
| Pre-pregnancy BMI | <25 kg/m^2^ | 467 (54.4) | 36 (57.1) | ref |
|  | 25-30 kg/m^2^ | 225 (26.2) | 14 (22.2) | 0.87 (0.45-1.66) |
|  | >30 kg/m^2^ | 132 (15.4) | 11 (17.5) | 1.15 (0.56-2.36) |
| Household income | <$20,000 | 328 (38.2) | 26 (41.3) | ref |
|  | $20,000-$40,000 | 225 (26.2) | 19 (30.2) | 1.02 (0.51-2.06) |
|  | >$40,000 | 177 (20.6) | 13 (20.6) | 0.83 (0.37-1.88) |
| Education level | <High school | 59 (6.9) | 5 (7.9) | ref |
|  | High school/equivalent | 109 (12.7) | 5 (7.9) | 0.50 (0.14-1.86) |
|  | Some college/technical | 308 (35.9) | 18 (28.6) | 0.64 (0.22-1.91) |
|  | College degree or higher | 372 (43.4) | 34 (54.0) | 1.17 (0.37-3.70) |
| Employment status | Unemployed | 314 (36.6) | 20 (31.7) | ref |
|  | Employed | 531 (61.9) | 43 (68.3) | 0.95 (0.50-1.81) |
| Marital status | Single | 177 (20.6) | 12 (19.0) | ref |
|  | Married/cohabitating | 673 (78.4) | 51 (81.0) | 1.04 (0.52-2.09) |
| Smoking in pregnancy | No | 833 (97.1) | 61 (96.8) | ref |
|  | Yes | 16 (1.9) | 2 (3.2) | 2.01 (0.42-9.73) |
| Alcohol use in pregnancy | No | 795 (92.7) | 58 (92.1) | ref |
|  | Yes | 51 (5.9) | 5 (7.9) | 1.33 (0.50-3.59) |
| Gravidity | 0-1 previous pregnancy | 337 (39.3) | 37 (58.7) | ref |
|  | >1 previous pregnancy | 512 (59.7) | 26 (41.3) | 0.55 (0.19-1.61) |
| Parity | No previous births | 407 (47.4) | 41 (65.1) | ref |
|  | ≥1 previous birth | 442 (51.5) | 22 (34.9) | 0.86 (0.28-2.64) |
| Mode of delivery | Vaginal | 459 (53.5) | 32 (50.8) | ref |
|  | C-section | 382 (44.5) | 30 (47.6) | 0.99 (0.58-1.69) |
| Infant sex | Male | 434 (50.6) | 36 (57.1) | ref |
|  | Female | 406 (47.3) | 26 (41.3) | 0.77 (0.45-1.32) |
| Season of delivery | November-June | 577 (67.2) | 41 (65.1) | ref |
|  | July-October | 281 (32.8) | 22 (34.9) | 1.18 (0.68-2.03) |
| Year of delivery | 2011 | 21 (2.4) | 1 (1.6) | 0.39 (0.05-3.19) |
|  | 2012 | 138 (16.1) | 11 (17.5) | 0.69 (0.29-1.66) |
|  | 2013 | 177 (20.6) | 8 (12.7) | 0.42 (0.16-1.07) |
|  | 2014 | 116 (13.5) | 12 (19.0) | ref |
|  | 2015 | 154 (17.9) | 7 (11.1) | 0.35 (0.13-0.95) |
|  | 2016 | 183 (21.3) | 17 (27.0) | 0.70 (0.31-1.58) |
|  | 2017 | 69 (8.0) | 7 (11.1) | 0.82 (0.30-2.29) |

^1^Models mutually adjusted for all covariates.

| **S2 File Table F. Odds ratios (OR) and 95% confidence intervals (CI) for small for gestational age (SGA) birth compared to appropriate for gestational age birth (AGA) in association with demographic and pregnancy characteristics in the PROTECT cohort 2011-2017.** | | | | |
| --- | --- | --- | --- | --- |
| **Characteristic** |  | AGA: N (%) | SGA: N (%) | OR (95% CI) |
| Age at enrollment | 18-24 | 311 (38.9) | 42 (43.8) | ref |
|  | 25-29 | 243 (30.4) | 27 (28.1) | 0.82 (0.49-1.37) |
|  | 30-34 | 165 (20.7) | 17 (17.7) | 0.76 (0.42-1.38) |
|  | ≥35 | 79 (9.9) | 10 (10.4) | 0.94 (0.45-1.95) |
| Pre-pregnancy BMI | <25 kg/m^2^ | 434 (54.3) | 55 (57.3) | ref |
|  | 25-30 kg/m^2^ | 205 (25.7) | 22 (22.9) | 0.85 (0.50-1.43) |
|  | >30 kg/m^2^ | 126 (15.8) | 14 (14.6) | 0.88 (0.47-1.63) |
| Household income | <$20,000 | 325 (40.7) | 41 (42.7) | ref |
|  | $20,000-$40,000 | 201 (25.2) | 26 (27.1) | 1.03 (0.61-1.73) |
|  | >$40,000 | 157 (19.6) | 20 (20.8) | 1.01 (0.57-1.78) |
| Education level | <High school | 59 (7.4) | 8 (8.3) | ref |
|  | High school/equivalent | 108 (13.5) | 9 (9.4) | 0.61 (0.23-1.68) |
|  | Some college/technical | 280 (35.0) | 37 (38.5) | 0.97 (0.43-2.20) |
|  | College degree or higher | 342 (42.8) | 40 (41.7) | 0.86 (0.38-1.93) |
| Employment status | Unemployed | 297 (37.2) | 35 (36.5) | ref |
|  | Employed | 490 (61.3) | 58 (60.4) | 1.00 (0.64-1.57) |
| Marital status | Single | 167 (20.9) | 16 (16.7) | ref |
|  | Married/cohabitating | 625 (78.2) | 78 (81.3) | 1.30 (0.74-2.29) |
| Smoking in pregnancy | No | 780 (97.6) | 89 (92.7) | ref |
|  | Yes | 11 (1.4) | 5 (5.2) | 3.98 (1.35-11.73) |
| Alcohol use in pregnancy | No | 739 (92.5) | 89 (92.7) | ref |
|  | Yes | 49 (6.1) | 5 (5.2) | 0.85 (0.33-2.18) |
| Gravidity | 0-1 previous pregnancy | 323 (40.4) | 47 (49.0) | ref |
|  | >1 previous pregnancy | 468 (58.6) | 47 (49.0) | 0.69 (0.45-1.06) |
| Parity | No previous births | 383 (47.9) | 55 (57.3) | ref |
|  | ≥1 previous birth | 408 (51.1) | 39 (40.6) | 0.67 (0.43-1.03) |
| Mode of delivery | Vaginal | 425 (53.2) | 55 (57.3) | ref |
|  | C-section | 374 (46.8) | 41 (42.7) | 0.85 (0.55-1.30) |
| Infant sex | Male | 426 (53.3) | 50 (52.1) | ref |
|  | Female | 373 (46.7) | 46 (47.9) | 1.05 (0.69-1.61) |
| Season of delivery | November-June | 528 (66.1) | 74 (77.1) | ref |
|  | July-October | 271 (33.9) | 22 (22.9) | 0.58 (0.35-0.95) |
| Year of delivery | 2011 | 18 (2.3) | 4 (4.2) | 2.26 (0.65-7.88) |
|  | 2012 | 141 (17.6) | 10 (10.4) | 0.72 (0.30-1.76) |
|  | 2013 | 165 (20.7) | 16 (16.7) | 0.99 (0.44-2.21) |
|  | 2014 | 112 (14.0) | 11 (11.5) | ref |
|  | 2015 | 143 (17.9) | 20 (20.8) | 1.42 (0.66-3.09) |
|  | 2016 | 167 (20.9) | 27 (28.1) | 1.65 (0.78-3.45) |
|  | 2017 | 53 (6.6) | 8 (8.3) | 1.54 (0.58-4.04) |

| **S2 File Table G. Odds ratios (OR) and 95% confidence intervals (CI) for large for gestational age (LGA) birth compared to appropriate for gestational age birth (AGA) in association with demographic and pregnancy characteristics in the PROTECT cohort 2011-2017.** | | | | |
| --- | --- | --- | --- | --- |
| **Characteristic** |  | AGA: N (%) | LGA: N (%) | OR (95% CI) |
| Age at enrollment | 18-24 | 311 (38.9) | 23 (23.5) | ref |
|  | 25-29 | 243 (30.4) | 40 (40.8) | 2.23 (1.30-3.82) |
|  | 30-34 | 165 (20.7) | 20 (20.4) | 1.64 (0.87-3.07) |
|  | ≥35 | 79 (9.9) | 15 (15.3) | 2.57 (1.28-5.15) |
| Pre-pregnancy BMI | <25 kg/m^2^ | 434 (54.3) | 44 (44.9) | ref |
|  | 25-30 kg/m^2^ | 205 (25.7) | 33 (33.7) | 1.59 (0.98-2.57) |
|  | >30 kg/m^2^ | 126 (15.8) | 18 (18.4) | 1.41 (0.79-2.52) |
| Household income | <$20,000 | 325 (40.7) | 34 (34.7) | ref |
|  | $20,000-$40,000 | 201 (25.2) | 23 (23.5) | 1.09 (0.63-1.91) |
|  | >$40,000 | 157 (19.6) | 29 (29.6) | 1.77 (1.04-3.00) |
| Education level | <High school | 59 (7.4) | 7 (7.1) | ref |
|  | High school/equivalent | 108 (13.5) | 9 (9.2) | 0.70 (0.25-1.98) |
|  | Some college/technical | 280 (35.0) | 33 (33.7) | 0.99 (0.42-2.35) |
|  | College degree or higher | 342 (42.8) | 49 (50.0) | 1.21 (0.52-2.79) |
| Employment status | Unemployed | 297 (37.2) | 33 (33.7) | ref |
|  | Employed | 490 (61.3) | 65 (66.3) | 1.19 (0.77-1.86) |
| Marital status | Single | 167 (20.9) | 14 (14.3) | ref |
|  | Married/cohabitating | 625 (78.2) | 84 (85.7) | 1.60 (0.89-2.90) |
| Smoking in pregnancy | No | 780 (97.6) | 97 (99.0) | ref |
|  | Yes | 11 (1.4) | 1 (1.0) | 0.73 (0.09-5.72) |
| Alcohol use in pregnancy | No | 739 (92.5) | 89 (90.8) | ref |
|  | Yes | 49 (6.1) | 8 (8.2) | 1.36 (0.62-2.96) |
| Gravidity | 0-1 previous pregnancy | 323 (40.4) | 31 (31.6) | ref |
|  | >1 previous pregnancy | 468 (58.6) | 67 (68.4) | 1.49 (0.95-2.34) |
| Parity | No previous births | 383 (47.9) | 39 (39.8) | ref |
|  | ≥1 previous birth | 408 (51.1) | 59 (60.2) | 1.42 (0.93-2.18) |
| Mode of delivery | Vaginal | 425 (53.2) | 48 (49.0) | ref |
|  | C-section | 374 (46.8) | 50 (51.0) | 1.18 (0.78-1.80) |
| Infant sex | Male | 426 (53.3) | 48 (49.0) | ref |
|  | Female | 373 (46.7) | 50 (51.0) | 1.19 (0.78-1.81) |
| Season of delivery | November-June | 528 (66.1) | 65 (66.3) | ref |
|  | July-October | 271 (33.9) | 33 (33.7) | 0.99 (0.63-1.54) |
| Year of delivery | 2011 | 18 (2.3) | 4 (4.1) | 1.78 (0.53-6.01) |
|  | 2012 | 141 (17.6) | 15 (15.3) | 0.85 (0.39-1.84) |
|  | 2013 | 165 (20.7) | 12 (12.2) | 0.58 (0.26-1.30) |
|  | 2014 | 112 (14.0) | 14 (14.3) | ref |
|  | 2015 | 143 (17.9) | 15 (15.3) | 0.84 (0.39-1.81) |
|  | 2016 | 167 (20.9) | 26 (26.5) | 1.25 (0.62-2.49) |
|  | 2017 | 53 (6.6) | 12 (12.2) | 1.81 (0.78-4.19) |

| **S2 File Table H. Adjusted^1^ odds ratios (OR) and 95% confidence intervals (CI) for small for gestational age (SGA) birth compared to appropriate for gestational age birth (AGA) in association with demographic and pregnancy characteristics in the PROTECT cohort 2011-2017.** | | | | |
| --- | --- | --- | --- | --- |
| **Characteristic** |  | AGA: N (%) | SGA: N (%) | OR (95% CI) |
| Age at enrollment | 18-24 | 311 (38.9) | 42 (43.8) | ref |
|  | 25-29 | 243 (30.4) | 27 (28.1) | 0.78 (0.44-1.40) |
|  | 30-34 | 165 (20.7) | 17 (17.7) | 0.79 (0.40-1.54) |
|  | ≥35 | 79 (9.9) | 10 (10.4) | 1.02 (0.45-2.28) |
| Pre-pregnancy BMI | <25 kg/m^2^ | 434 (54.3) | 55 (57.3) | ref |
|  | 25-30 kg/m^2^ | 205 (25.7) | 22 (22.9) | 0.87 (0.51-1.48) |
|  | >30 kg/m^2^ | 126 (15.8) | 14 (14.6) | 0.97 (0.51-1.84) |
| Household income | <$20,000 | 325 (40.7) | 41 (42.7) | ref |
|  | $20,000-$40,000 | 201 (25.2) | 26 (27.1) | 1.15 (0.62-2.15) |
|  | >$40,000 | 157 (19.6) | 20 (20.8) | 1.30 (0.64-2.67) |
| Education level | <High school | 59 (7.4) | 8 (8.3) | ref |
|  | High school/equivalent | 108 (13.5) | 9 (9.4) | 0.58 (0.21-1.61) |
|  | Some college/technical | 280 (35.0) | 37 (38.5) | 0.84 (0.36-1.99) |
|  | College degree or higher | 342 (42.8) | 40 (41.7) | 0.71 (0.28-1.80) |
| Employment status | Unemployed | 297 (37.2) | 35 (36.5) | ref |
|  | Employed | 490 (61.3) | 58 (60.4) | 0.90 (0.53-1.54) |
| Marital status | Single | 167 (20.9) | 16 (16.7) | ref |
|  | Married/cohabitating | 625 (78.2) | 78 (81.3) | 1.39 (0.75-2.57) |
| Smoking in pregnancy | No | 780 (97.6) | 89 (92.7) | ref |
|  | Yes | 11 (1.4) | 5 (5.2) | 5.61 (1.73-18.18) |
| Alcohol use in pregnancy | No | 739 (92.5) | 89 (92.7) | ref |
|  | Yes | 49 (6.1) | 5 (5.2) | 0.79 (0.30-2.10) |
| Gravidity | 0-1 previous pregnancy | 323 (40.4) | 47 (49.0) | ref |
|  | >1 previous pregnancy | 468 (58.6) | 47 (49.0) | 0.93 (0.41-2.09) |
| Parity | No previous births | 383 (47.9) | 55 (57.3) | ref |
|  | ≥1 previous birth | 408 (51.1) | 39 (40.6) | 0.69 (0.30-1.58) |
| Mode of delivery | Vaginal | 425 (53.2) | 55 (57.3) | ref |
|  | C-section | 374 (46.8) | 41 (42.7) | 0.82 (0.53-1.27) |
| Infant sex | Male | 426 (53.3) | 50 (52.1) | ref |
|  | Female | 373 (46.7) | 46 (47.9) | 1.13 (0.73-1.74) |
| Season of delivery | November-June | 528 (66.1) | 74 (77.1) | ref |
|  | July-October | 271 (33.9) | 22 (22.9) | 0.61 (0.37-1.01) |
| Year of delivery | 2011 | 18 (2.3) | 4 (4.2) | 2.56 (0.71-9.17) |
|  | 2012 | 141 (17.6) | 10 (10.4) | 0.79 (0.32-1.95) |
|  | 2013 | 165 (20.7) | 16 (16.7) | 0.94 (0.42-2.13) |
|  | 2014 | 112 (14.0) | 11 (11.5) | ref |
|  | 2015 | 143 (17.9) | 20 (20.8) | 1.40 (0.63-3.10) |
|  | 2016 | 167 (20.9) | 27 (28.1) | 1.55 (0.71-3.36) |
|  | 2017 | 53 (6.6) | 8 (8.3) | 1.40 (0.52-3.81) |

^1^Models mutually adjusted for all covariates.

| **S2 File Table I. Adjusted^1^ odds ratios (OR) and 95% confidence intervals (CI) for large for gestational age (LGA) birth compared to appropriate for gestational age birth (AGA) in association with demographic and pregnancy characteristics in the PROTECT cohort 2011-2017.** | | | | |
| --- | --- | --- | --- | --- |
| **Characteristic** |  | AGA: N (%) | LGA: N (%) | OR (95% CI) |
| Age at enrollment | 18-24 | 311 (38.9) | 23 (23.5) | ref |
|  | 25-29 | 243 (30.4) | 40 (40.8) | 2.40 (1.30-4.44) |
|  | 30-34 | 165 (20.7) | 20 (20.4) | 1.79 (0.86-3.70) |
|  | ≥35 | 79 (9.9) | 15 (15.3) | 2.60 (1.18-5.74) |
| Pre-pregnancy BMI | <25 kg/m^2^ | 434 (54.3) | 44 (44.9) | ref |
|  | 25-30 kg/m^2^ | 205 (25.7) | 33 (33.7) | 1.56 (0.95-2.54) |
|  | >30 kg/m^2^ | 126 (15.8) | 18 (18.4) | 1.32 (0.73-2.40) |
| Household income | <$20,000 | 325 (40.7) | 34 (34.7) | ref |
|  | $20,000-$40,000 | 201 (25.2) | 23 (23.5) | 1.12 (0.58-2.16) |
|  | >$40,000 | 157 (19.6) | 29 (29.6) | 1.79 (0.90-3.56) |
| Education level | <High school | 59 (7.4) | 7 (7.1) | ref |
|  | High school/equivalent | 108 (13.5) | 9 (9.2) | 0.66 (0.23-1.89) |
|  | Some college/technical | 280 (35.0) | 33 (33.7) | 0.86 (0.35-2.16) |
|  | College degree or higher | 342 (42.8) | 49 (50.0) | 0.96 (0.36-2.53) |
| Employment status | Unemployed | 297 (37.2) | 33 (33.7) | ref |
|  | Employed | 490 (61.3) | 65 (66.3) | 0.99 (0.58-1.68) |
| Marital status | Single | 167 (20.9) | 14 (14.3) | ref |
|  | Married/cohabitating | 625 (78.2) | 84 (85.7) | 1.39 (0.74-2.59) |
| Smoking in pregnancy | No | 780 (97.6) | 97 (99.0) | ref |
|  | Yes | 11 (1.4) | 1 (1.0) | 0.88 (0.11-7.16) |
| Alcohol use in pregnancy | No | 739 (92.5) | 89 (90.8) | ref |
|  | Yes | 49 (6.1) | 8 (8.2) | 1.57 (0.70-3.50) |
| Gravidity | 0-1 previous pregnancy | 323 (40.4) | 31 (31.6) | ref |
|  | >1 previous pregnancy | 468 (58.6) | 67 (68.4) | 1.15 (0.48-2.74) |
| Parity | No previous births | 383 (47.9) | 39 (39.8) | ref |
|  | ≥1 previous birth | 408 (51.1) | 59 (60.2) | 1.22 (0.53-2.81) |
| Mode of delivery | Vaginal | 425 (53.2) | 48 (49.0) | ref |
|  | C-section | 374 (46.8) | 50 (51.0) | 1.13 (0.73-1.74) |
| Infant sex | Male | 426 (53.3) | 48 (49.0) | ref |
|  | Female | 373 (46.7) | 50 (51.0) | 1.18 (0.77-1.80) |
| Season of delivery | November-June | 528 (66.1) | 65 (66.3) | ref |
|  | July-October | 271 (33.9) | 33 (33.7) | 1.01 (0.64-1.58) |
| Year of delivery | 2011 | 18 (2.3) | 4 (4.1) | 1.70 (0.49-5.91) |
|  | 2012 | 141 (17.6) | 15 (15.3) | 0.87 (0.40-1.90) |
|  | 2013 | 165 (20.7) | 12 (12.2) | 0.59 (0.26-1.34) |
|  | 2014 | 112 (14.0) | 14 (14.3) | ref |
|  | 2015 | 143 (17.9) | 15 (15.3) | 0.85 (0.39-1.85) |
|  | 2016 | 167 (20.9) | 26 (26.5) | 1.29 (0.63-2.65) |
|  | 2017 | 53 (6.6) | 12 (12.2) | 1.72 (0.72-4.07) |

^1^Models mutually adjusted for all covariates.
